# A central circadian oscillator confers defense heterosis in hybrids without growth vigor costs

**DOI:** 10.1038/s41467-021-22268-z

**Published:** 2021-04-19

**Authors:** Li Yang, Pengtao Liu, Xuncheng Wang, Aolin Jia, Diqiu Ren, Yaru Tang, Yaqi Tang, Xing Wang Deng, Guangming He

**Affiliations:** 1 School of Advanced Agricultural Sciences and School of Life Sciences, State Key Laboratory of Protein and Plant Gene Research, Peking-Tsinghua Center for Life Sciences, Peking University, Beijing, China; 2 Department of Plant Pathology, China Agricultural University, Beijing, China; 3 Peking University-Southern University of Science and Technology Institute of Plant and Food Science, Department of Biology, Southern University of Science and Technology, Shenzhen, China

**Keywords:** Circadian rhythms in plants, Plant hybridization, Plant immunity

## Abstract

Plant immunity frequently incurs growth penalties, which known as the trade-off between immunity and growth. Heterosis, the phenotypic superiority of a hybrid over its parents, has been demonstrated for many traits but rarely for disease resistance. Here, we report that the central circadian oscillator, *CCA1*, confers heterosis for bacterial defense in hybrids without growth vigor costs, and it even significantly enhances the growth heterosis of hybrids under pathogen infection. The genetic perturbation of *CCA1* abrogated heterosis for both defense and growth in hybrids. Upon pathogen attack, the expression of *CCA1* in F_1_ hybrids is precisely modulated at different time points during the day by its rhythmic histone modifications. Before dawn of the first infection day, epigenetic activation of *CCA1* promotes an elevation of salicylic acid accumulation in hybrids, enabling heterosis for defense. During the middle of every infection day, diurnal epigenetic repression of *CCA1* leads to rhythmically increased chlorophyll synthesis and starch metabolism in hybrids, effectively eliminating the immunity-growth heterosis trade-offs in hybrids.

## Abstract (web summary)

There is frequently a trade-off between plant immunity and growth. Here the authors show that the epigenetic control of CCA1, encoding a core component of the circadian oscillator, simultaneously promotes heterosis for both defense and growth in hybrids under pathogen invasion.

## Introduction

Hybrids often show phenotypic superiority for many traits, such as growth rate, biomass, and stress tolerance, than their parents, a phenomenon known as hybrid vigor or heterosis^1^, which has been broadly used in crop breeding and has made an enormous contribution to world food production^2^. In contrast to the widespread utilization of heterosis in crop production over a century, our understanding of its molecular basis is still rudimentary^2–5^. Recent studies have identified circadian regulatory genes^6^ and stress response genes^7–9^ that contribute to growth heterosis in *Arabidopsis* hybrids, and enhanced salicylic acid (SA) biosynthesis contributes to bacterial defense heterosis in *Arabidopsis* hybrids^10^. Another study reported that a single overdominant gene, *SINGLE FLOWER TRUSS*, is responsible for fruit yield heterosis in tomato^11^. In addition, genome-wide association studies have identified a small number of genomic loci that contribute to yield heterosis in rice hybrids^12,13^ and growth heterosis in *Arabidopsis* hybrids^14^, respectively. Moreover, several studies suggesting that both transcriptional and epigenetic variations (including DNA methylation, small RNA, and histone modifications) may play a role in the molecular mechanisms of growth heterosis^3,4,9,15–18^. Despite several studies have shed light on the potential molecular mechanisms of heterosis in plants, most have focused on heterosis for growth but rarely for heterosis for disease resistance. And, most of these studies were conducted on several candidate genes or multiple biological pathways, only a few major or single genetic factors were identified that clearly contribute to heterosis.

Plant diseases cause enormous yield losses and threaten global food security. The use of highly resistant cultivars can effectively control plant disease; however, mounting a defense response frequently incurs yield penalties in plants^19,20^, which is known as the trade-off between defense and growth or yield. The cost of resistance was first reported in the early 1960s for late blight disease of potato (*Solanum tuberosum*)^21^ and has since been documented in other crops and *Arabidopsis*^20,22^. For examples, utilization of resistance (*R*) genes, such as *Wsm1* for wheat (*Triticum aestivum*) streak mosaic virus and *RPM1*, *RPS5* for resistance to *Pseudomonas syringae* in *Arabidopsis*, is associated with a mean yield reduction of 21%^23^ and 5–10%^24^, respectively. In addition to R proteins, the *Arabidopsis* transcription factors TBF1 and WRKY45 enhance immunity but inhibit plant growth^25,26^. Conversely, the bHLH transcription factor HBI1 can promote plant growth but suppress immunity^27,28^. Plants have evolved several mechanisms to reduce the magnitude of immunity-growth trade-offs. In rice, a better balance between growth and immunity was obtained by a natural allele of the *Broad-Spectrum Resistance-Digu 1* (*Bsr-d1*) transcription factor gene^29^; a single gene *Ideal Plant Architecture 1* (*IPA1*)^30^; a nucleotide-binding oligomerization domain-like receptor (NLR) pair, *Pyricularia*-*Gumei* *Resistant* and *Pyricularia*-*Gumei*  *Susceptible* (*PigmR* and *PigmS*)^31^; or an artificial, pathogen-inducible cassette containing *Nonexpressor* *of* *Pathogenesis-Related genes 1* (*NPR1*) or *snc1* (*suppressor of npr1-1, constitutive 1*)^32^. Although the series of studies described above have explored the immunity-growth trade-offs in wild accessions of plants, whether and how higher plants reconcile defense and growth heterosis in hybrids, the two antagonistic biological processes, remained unexplored until now. This line of study is of substantial importance for agriculture, since it may provide an avenue to develop hybrids that have strong, durable disease resistance without yield penalties.

In this study, we identified the central circadian oscillator, *CCA1*, which confers significant heterosis for disease resistance in F_1_ hybrids without growth vigor penalties upon pathogen invasion, by precisely enhancing the ability to resist disease and the growth of hybrids at different time points of a day and also on different infection days. Before dawn of the first infection day, epigenetic activation of *CCA1* resulted in a higher burst of SA in F_1_ hybrids than that in parents, which resulted in significant heterosis for defense in hybrids. In the middle of every infection day, diurnal epigenetic repression of *CCA1* led to rhythmic enhancement of growth-related pathways in F_1_ hybrids, which dramatically recovered the growth consumption caused by higher levels of defense in hybrids. By this time-scheduled regulation strategy, hybrids gain advantages from the control of circadian-mediated physiological and metabolic pathways, leading to better reallocation of limited resources to ensure significantly enhanced ability to resist disease with the least growth costs.

## Results

### *CCA1* confers significant heterosis for defense in hybrids

**Fig. 1:**
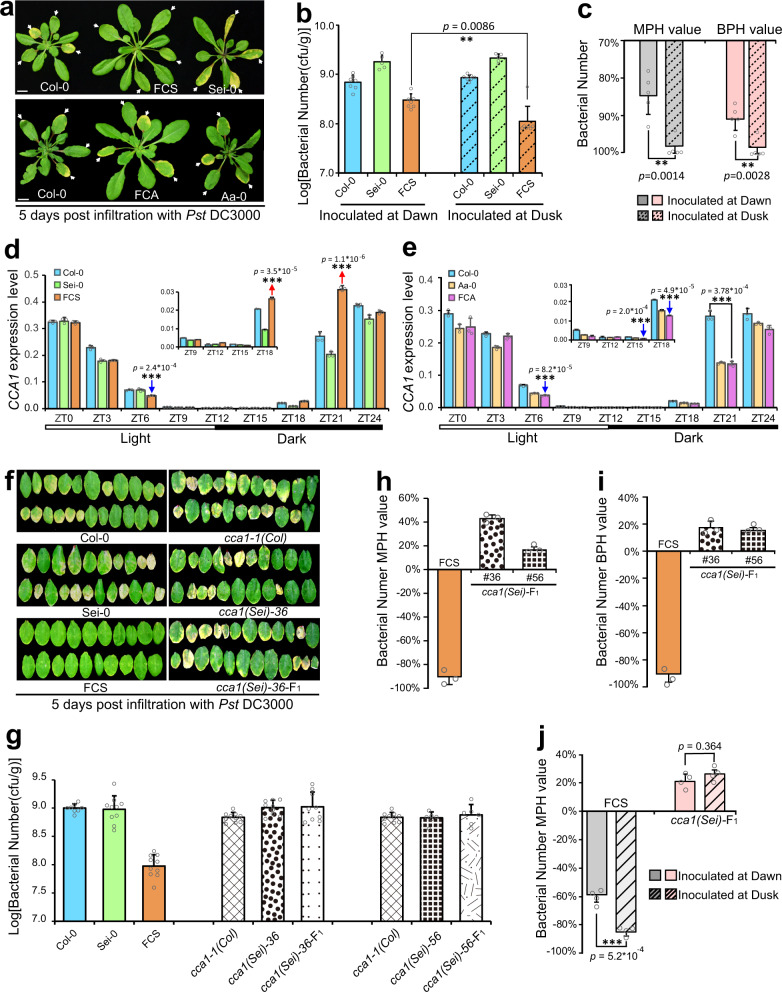
*CCA1* confers significant heterosis for defense in hybrids. **a** Phenotypes of F_1_ hybrids and parents 5 dpi with *Pst* DC3000. Arrows show the leaves inoculated with pathogen. The scale represents 1 cm. **b** Bacterial titer (log10) of the F_1_ hybrids and parents 5 dpi with *Pst* DC3000 at dawn and at dusk, respectively. **c** Mid-parent heterosis (MPH) and best-parent heterosis (BPH) values of FCS hybrids calculated by the bacterial number of 5 dpi at dawn and at dusk, respectively. Data are shown as the mean ± SD. Quantitative RT-PCR analysis of the *CCA1*’s expression level in the F_1_ hybrids and parents of Col-0 × Sei-0 (**d**) and Col-0 × Aa-0 (**e**) in a 24-h period (12-h light/12-h dark cycles) starting at dawn (ZT0) with *Pst* DC3000 infiltration. Data are shown as the mean ± SD (*n* = 3, *n* indicates biological replicates). Arrows indicate significant upregulation (red) and downregualtion (blue) of *CCA1* in F_1_ hybrids compared with that in both parents. **f** Leaf phenotypes of wild-type and *CCA1*-mutated F_1_ hybrids and parents at 5 dpi with *Pst* DC3000. **g** Bacterial titer (log10) of the wild-type and *CCA1*-mutated F_1_ hybrids and parents at 5 dpi with *Pst* DC3000. MPH value (**h**) and BPH value (**i**) of wild-type and *CCA1*-mutated F_1_ hybrids calculated by bacterial number at 5 dpi. Data are shown as the mean ± SD. **j** MPH value of wild-type and *CCA1*-mutated F_1_ hybrids calculated by bacterial number (5 dpi) at dawn and dusk, respectively. Data are shown as the mean ± SD. Bacterial growth in **b** and **g** is indicated as the mean values of viable bacteria per gram of leaf tissue ± SD (*n* = 6, *n* indicates biological replicates). Both MPH and BPH values of FCS represented by the bacterial number were negative in **h**–**j** due to the bacterial number in FCS was significantly less than that in its parents. The results in **b**–**j** are representative of three independent experiments, with measurements taken from independent samples grown and processed at different times. dpi: days post infiltration. **p value < 0.01; ***p value < 0.001 (two-tailed Student’s *t* test).

Our previous study identified an *Arabidopsis* hybrid Col-0 × Sei-0 (designated FCS) that shows significant heterosis for biotrophic bacterial defense (Fig. 1a), which was attributed to an increase in the accumulation of SA in F_1_ hybrids compared with both parents when the pathogen invaded^10^. To explore other factors involved in the control of heterosis for defense in FCS hybrids, we analyzed the promoter regions of previously identified above-high parent differentially expressed genes (DEGs) in FCS at 1, 2, and 3 days after infiltration with *Pst* DC3000 (*n* = 1326, see Supplementary Data 1)^10^. Interestingly, the “evening element” (AAAATATCT) was the most significantly (*p* = 2.66 × 10^−15^) enriched motif in the promoter regions of these above-high parent DEGs. Moreover, these evening element-containing above-high parent DEGs are significantly enriched in defense-related and circadian rhythm pathways (Supplementary Table 1 and Fig. 1), which imply that these DEGs are probably the targets of CCA1 or LHY^33,34^, and *CCA1* or *LHY* mediated circadian clock might contribute to heterosis for bacterial defense by a significantly greater upregulation of the genes involved in defense in hybrids than both parents when the plants are attacked by a pathogen.

To verify this hypothesis, we first inoculated parents and hybrids with *Pst* DC3000 not only at the normal “dawn” infection time but also at “dusk.” We found that if Col-0, Sei-0, and their hybrids were inoculated at dusk, when infection was unexpected, significantly increased levels of susceptibility were observed in both Col-0 and Sei-0 but not in FCS. As opposed to its parents, FCS became more resistant when pathogens were inoculated at “dusk” than at “dawn” (Fig. 1b). This result indicates that hybrids are more adaptable to unexpected infection than their parents. Calculating the mid-parent heterosis (MPH) and better-parent heterosis (BPH) values by bacterial number at 5 days post infiltration (dpi) showed that the degree of heterosis for defense was obviously higher when inoculated at “dusk” than when inoculated at “dawn” (Fig. 1c), suggesting the involvement of the circadian clock in the regulation of heterosis for bacterial defense.

Next, we tested the expression level of *CCA1* in hybrids and parents grown under 12-h light/12-h dark photocycles every 3 h up to 24 h post infiltration (hpi) with or without *Pst* DC3000. *Pst* DC3000 was infiltrated at ZT0 (0 hpi, 9:00 a.m., the light is on at this time). Noticeably, when compared with that in their parents, the *CCA1*’s expression level at 21 hpi was significantly increased in FCS, while it was significantly decreased compared with mid-parent value (MPV) in another hybrid, Col-0 × Aa-0 (designated FCA), which showed no heterosis for bacterial defense (Fig. 1a, d, e). In addition, this above-high parent pattern of expression of *CCA1* in FCS only occurred when the pathogen invaded, which was not detected in the noninfiltrated condition (Fig. 1d, e and Supplementary Fig. 2). These results suggested that the above-high parent expression pattern of *CCA1* at 21 hpi might be responsible for significant heterosis for defense in FCS hybrids, and *CCA1* tends to activate defense responses before dawn of the first infection day (21 hpi) more strongly in FCS hybrids than in their parents; this time precisely precedes the peak of expression of SA biosynthetic genes at 24 hpi in the FCS hybrids^10^.

To confirm the involvement of *CCA1* in heterosis for defense, we generated two CRISPR/CAS9-based knockout lines of *CCA1* in the Sei-0 background, each manifesting significantly decreased transcription and null translation of *CCA1* (Supplementary Fig. 3). We obtained hybrids that were deficient in *CCA1* by crossing *CCA1* mutants in a Col-0 background (*cca1-1*) with those in a Sei-0 background (*cca1(Sei)-36,56*) and inoculated them with *Pst* DC3000. We noted that the *CCA1*-mutated F_1_ hybrids had completely lost heterosis for defense when evaluated via either phenotype or bacterial number at 5 dpi or the expression of *PR1* (a marker gene that functions downstream of SA^35^) at 24 hpi (Fig. 1f, g and Supplementary Fig. 4). The MPH and BPH values of the bacterial number at 5 dpi implied that the *CCA1*-mutated F_1_ hybrids were more susceptible than the *CCA1*-mutated parents, which is opposite to the wild-type FCS phenotype (Fig. 1h, i). Furthermore, the phenomenon that heterosis for defense was more obvious when inoculated at “dusk” than when inoculated at “dawn” disappeared in the hybrids in which *CCA1* had been mutated (Fig. 1j), implying that circadian-regulated heterosis for defense is dependent on *CCA1*. Moreover, heterosis for defense was also completely abolished in the *CCA1*-mutated hybrids that were generated from CRISPR/CAS9-based knockout lines in both the Col-0 and Sei-0 backgrounds (Supplementary Fig. 5). Unlike *CCA1*, the mutation of the other two transcription factor genes, *LATE ELONGATED HYPOCOTYL* (*LHY*) and *TIMING OF CAB2 EXPRESSION 1* (*TOC1*), which were also involved in the core loop of eukaryotic circadian clocks^36,37^, did not change the degree of defense heterosis (Supplementary Figs. 6 and 7), and the phenomenon that heterosis for defense was more obvious when inoculated at “dusk” than when inoculated at “dawn” was also not influenced by the mutation of either *LHY* or *TOC1* (Supplementary Fig. 8). These data provide strong evidence that *CCA1* confers significant heterosis for defense in F_1_ hybrids.

Additional F_1_ hybrids and their parents (*n* = 18) were selected to test the *CCA1*’s expression level at 21 hpi and calculate the MPH value of F_1_ hybrids at 5 dpi (Supplementary Table 2). We found that in 13 of 18 hybrids (72.2%), the differential patterns of expression of *CCA1* at 21 hpi were consistent with the fact that whether or not these hybrids display heterosis for defense (the cycled F_1_ hybrids in Supplementary Fig. 9): five hybrids with an increased expression of *CCA1* at 21 hpi compared with MPV show heterosis for defense (calculated by the bacterial number at 5 dpi), which is similar to the FCS hybrid, and all eight hybrids with a decreased expression of *CCA1* compared with the MPV at 21 hpi show no heterosis for defense, which is similar to the FCA hybrid (Supplementary Fig. 9). These results imply that the above-high parent expression pattern of *CCA1* in a hybrid at 21 hpi contributed to whether this hybrid exhibits heterosis for defense upon pathogen attack. Altogether, these data suggest that *CCA1* is a common regulator that is essential for heterosis for defense in different *Arabidopsis* hybrids.

### *CCA1* confers defense heterosis by enhancing SA biosynthesis in hybrids

**Fig. 2:**
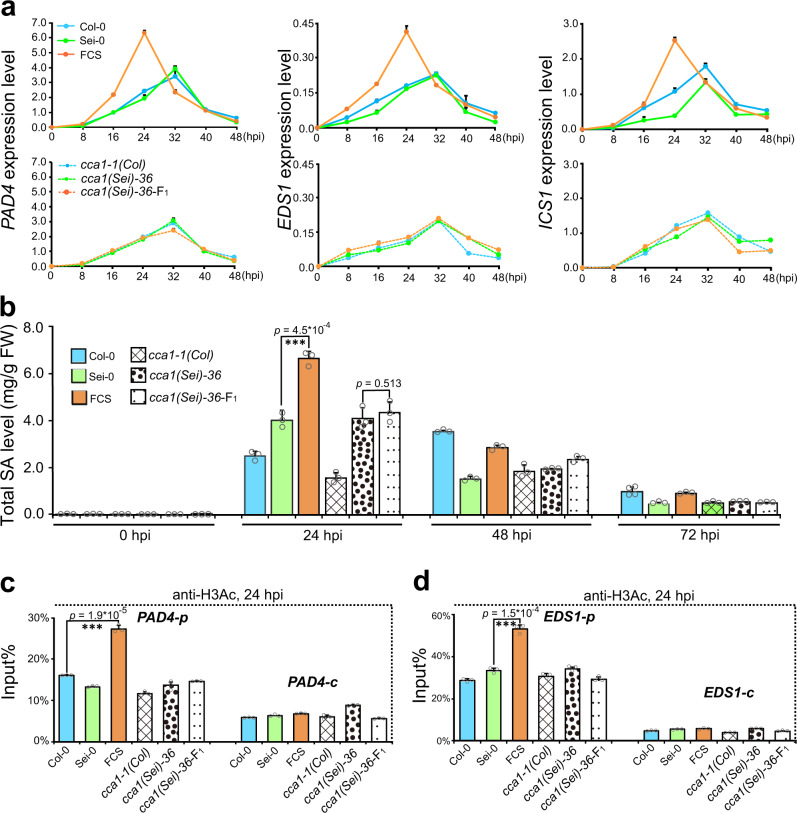
*CCA1* confers defense heterosis by enhancing salicylic acid biosynthesis in hybrids. **a** qPCR analyses of the expression level of *PAD4*, *EDS1*, and *ICS1* in Col-0 × Sei-0 and *CCA1*-mutated F_1_ hybrids and parents every 8 h post infiltration (hpi) up to 48 hpi. Data are standardized for the abundance of the *ACTIN2* transcript. Data are shown as the mean ± SD (*n* = 3, *n* indicates biological replicates). **b** Total SA level in Col-0 × Sei-0 and *CCA1*-mutated F_1_ hybrids and parents at 0, 24, 48, and 72 hpi with *Pst* DC3000 inoculation. Data are shown as the mean ± SD (*n* = 4, *n* indicates biological replicates). SA salicylic acid. ChIP-qPCR analyses of promoter fragments (*PAD4-p* and *EDS1-p*) and exon fragments (*PAD4-c* and *EDS1-c*) of *PAD4* (**c**) and *EDS1* (**d**) in Col-0 × Sei-0 and *CCA1*-mutated F_1_ hybrids and parents using an anti-H3Ac antibody at 24 hpi. ChIP values were normalized to their respective DNA inputs. The results are representative of three biological replicates, with measurements taken from independent samples grown and processed at different times. Data are shown as the mean ± SD (*n* = 3, *n* indicates biological replicates). ***p value < 0.001 (two-tailed Student’s *t* test).

To further explore how *CCA1* regulates heterosis for defense, we first examined the expression levels of SA biosynthetic genes in the wild-type and *CCA1*-mutated parents and hybrids, including the most important SA biosynthetic gene, *ICS1*^38,39^, the upstream transcription factor genes *CBP60g*, *SARD1*^40^, and their upstream regulators *EDS1*, *PAD4*^41–43^. We found that the expression level of all these SA biosynthetic genes in the *CCA1*-mutated hybrids did not increase significantly when compared with that in the *CCA1*-mutated parents, and the 8 h earlier peak of expression in the wild-type FCS compared with that in its parents was also eliminated in the *CCA1*-mutated hybrids (Fig. 2a and Supplementary Fig. 10a). Both the significant above-high parent and the earlier expression of SA biosynthetic genes in hybrids compared with that in the parents have been shown to be essential for heterosis for defense^10^. Consistent with the pattern of expression of the SA biosynthetic genes, more SA accumulated in the wild-type hybrid of FCS than in its parents at 24 hpi (*p* < 0.01, Student’s *t* test), which was not observed in the *CCA1*-mutated hybrids (Fig. 2b), indicating that *CCA1* is an essential regulator for the above-high parent accumulation of SA in the F_1_ hybrids.

It is notable that the mutation of *CCA1* specifically affects the above-high parent expression of SA biosynthetic genes and SA accumulation in F_1_ hybrid, but does not block SA biosynthesis itself in parents. Given that the increased histone H3 acetylation (H3Ac) level of key SA biosynthetic genes correlates closely with their above-high parent expression in infected hybrids^10^, we investigated the levels of H3Ac of key SA biosynthetic genes in *CCA1*-mutated and wild-type parents and hybrids. We found that the levels of H3Ac at the promoter regions of the genes tested were significantly increased in FCS relative to those in both parents at 24 hpi, but there was no difference in the levels of H3Ac between the *CCA1*-mutated F_1_ hybrids and their parents for all four genes (*PAD4*, *EDS1*, *CBP60g*, and *SARD1*) that are essential for SA biosynthesis (Fig. 2c, d and Supplementary Fig. 10b, c). Taken together, our results demonstrated that *CCA1* confers heterosis for defense by specifically triggering the above-high parent expression of SA biosynthetic genes in the F_1_ hybrids, possibly through the regulation of these genes’ histone modifications, such as H3Ac, which ultimately results in above-high parent accumulation of SA in hybrids.

### *CCA1* confers defense heterosis in hybrids without growth vigor costs

**Fig. 3:**
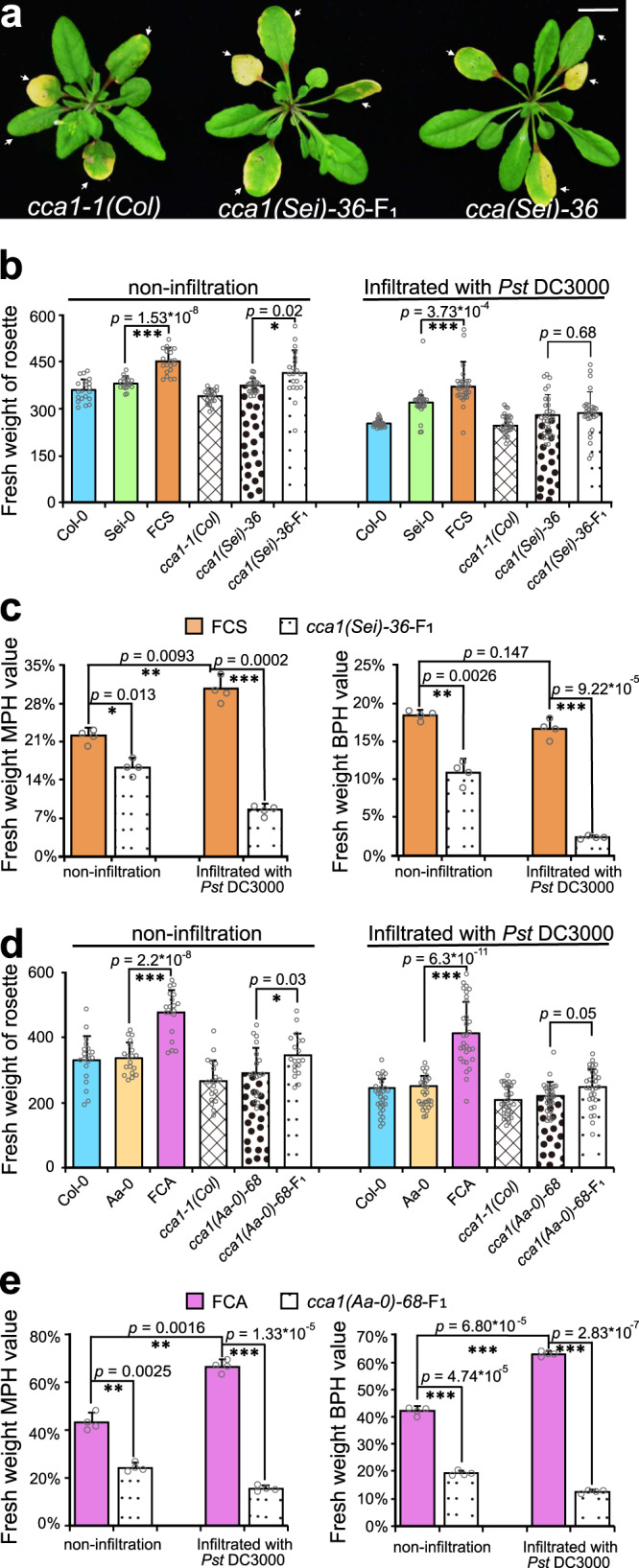
*CCA1* confers defense heterosis in hybrids without growth vigor costs. **a** Phenotypes of the whole rosette of the *CCA1*-mutated F_1_ hybrids and parents at 5 days post infiltration. Arrows show the four leaves of each plant inoculated with the pathogen. The scale represents 1 cm. **b**, **d** Fresh weight of the whole rosette of wild-type and *CCA1*-mutated hybrids and parents simultaneously with *Pst* DC3000 infiltration or not. 20-day-old (counted from the day the seedlings were transferred from MS plates to the soil) plants were infiltrated with *Pst* DC3000 or not infiltrated. The fresh weight of whole rosette was calculated 5 days later for both noninoculated and inoculated genotypes. Data are shown as the mean ± SD (*n* = 20 plants for each genotype without *Pst* DC3000 infiltration, and *n* = 30 plants for each genotype with *Pst* DC3000 infiltration). **c**, **e** MPH and BPH values of wild-type and *CCA1*-mutated hybrids calculated by whole rosette fresh weight at the same time without (noninfiltration) or with *Pst* DC3000 infiltration. Data are shown as the mean ± SD. The results in **c** and **e** are representative of three independent experiments, with measurements that were taken from independent samples grown and processed at different times. *p value < 0.05; **p value < 0.01; and ***p value < 0.001 (two-tailed Student’s *t* test).

Importantly, with pathogen infection, the wild-type hybrids FCS with remarkable defense heterosis did not display growth vigor penalties, and still showed evident heterosis for growth, as indicated by a significantly higher fresh/dry weight of the rosette or infiltrated leaves than that of its both parents at 5 dpi (Figs. 1a and 3b and Supplementary Fig. 11). Even more notably, the growth heterosis of FCS became more evident under disease pressure compared with that in the noninfiltration condition (without pathogen infection): the MPH value of rosette fresh weight in hybrids increased significantly from 21.9 to 30.4% (*p* value = 0.0093) after pathogen invasion, indicating that the hybrids can obviously reduce the growth consumption caused by immunity more effectively than their parents (Fig. 3c). However, in *CCA1*-mutated F_1_ hybrids, both the defense and growth vigor were almost abolished compared with the *CCA1*-mutated parents after pathogen inoculation (Figs. 1f, g and 3b). Moreover, the decline of growth vigor caused by the mutation of *CCA1* became more dramatic when pathogen invasion (with a 22% reduction of MPH and a 14.2% reduction of BPH) than that in noninfiltration condition (with a 5.7% reduction of MPH and a 7.5% reduction of BPH) (Fig. 3a–c). These results indicate that the heterosis for growth in the FCS hybrids was partially dependent on *CCA1* in noninfiltration condition, but totally dependent on *CCA1* following pathogen invasion, and that hybrids achieved significant defense heterosis without consuming growth vigor is completely owing to *CCA1*.

Another F_1_ hybrids, FCA, which crossed from Col-0 and Aa-0, also showed significant heterosis for growth after pathogen inoculation (Figs. 1a and 3d). Similar to that of FCS, the growth vigor of FCA became even more evident under pathogen pressure compared with that in noninfiltration condition: the MPH value of rosette fresh weight in the FCA hybrids increased significantly from 43 to 66% (*p* value = 0.0016) after pathogen infection. In contrast, the mutation of *CCA1*, which was mutated in both Col-0 and Aa-0 background, almost abolished the growth vigor of FCA upon pathogen invasion with a 51% reduction of both MPH and BPH, but it only significantly decreased the growth vigor of FCA in noninfiltration condition with a 19% reduction of MPH and a 23% reduction of BPH (Fig. 3e). This indicates that *CCA1* also plays an important role in the growth vigor of FCA hybrids in both noninfiltration or pathogen infection conditions and is exceptionally important under conditions of pathogen invasion. Unlike FCS, the defense ability of FCA did not differ significantly from that of its parents, and there is no evident heterosis for defense in the FCA hybrids at 5 dpi; in addition, the mutation of *CCA1* maintained no heterosis for defense in FCA, which is consistent with the lack of above-high parent pattern of expression of *CCA1* at 21 hpi in the FCA hybrids compared with their parents (Fig. 1e and Supplementary Figs. 2 and 12). Taken together, all these results suggested that *CCA1* confers significant defense heterosis in hybrids without growth vigor penalties and even dramatically promotes growth heterosis in hybrids under pathogen infection.

In addition, we calculated heterosis for growth (MPH values) by fresh and dry weight of the rosette 5 days after pathogen inoculation, and detected the *CCA1*’s expression level at 6 hpi in 14 hybrids and parents (Supplementary Table 3). We found that 13 F_1_ hybrids (with the exception of Ca-0-F_1_) exhibited heterosis for growth when calculated by either fresh or dry weight at 5 dpi. In addition, 9 of these 13 F_1_ hybrids (69.2%, the cycled F_1_ hybrids in Supplementary Fig. 13) displayed a below-low parent pattern of expression of *CCA1* at 6 hpi (Supplementary Fig. 13). These results implied that the differential pattern of expression of *CCA1* in hybrids at 6 hpi also contributes to whether or not these F_1_ hybrids exhibit heterosis for growth.

### *CCA1* eliminates defense-growth heterosis trade-offs in hybrids by a time-scheduled regulation strategy

Having confirmed that *CCA1* confers significant defense heterosis in hybrids without growth vigor costs upon pathogen invasion, we set out to explore how *CCA1* reconcile heterosis for defense and for growth, the two antagonistic biological processes, without trade-offs in the hybrids. It has been reported that plants with the *CCA1* mutation were significantly more susceptible to *P. syringae* than the wild-type^44^. Conversely, reducing the expression of *CCA1* can promote the accumulation of chlorophyll and starch, which increases the growth of plants^6^, indicating that *CCA1* exhibits opposite roles in plant immunity and growth. Corresponding to the opposite role of *CCA1* in immunity and growth, *CCA1* manifested below-low parent expression pattern of *CCA1* in FCS hybrids at 6 hpi, but converse to above-high parent expression pattern of *CCA1* in the FCS hybrids at 21 hpi, which is consistent with the role of *CCA1* in the significantly enhancement of both defense and growth heterosis of the FCS hybrids upon pathogen invasion. In addition, *CCA1*’s expression level decreased significantly in both the FCS and FCA hybrids compared with that in their parents at ZT6 weather or not they were inoculated with *Pst* DC3000 (Fig. 1d, e and Supplementary Fig. 2). Considering that both FCS and FCA showed significantly growth heterosis and the same below-low parent expression patterns of *CCA1* at ZT6 no matter inoculated with *Pst* DC3000 or not (Fig. 1a, d, e and Supplementary Fig. 2) and that plants accumulate energy and materials required for growth during the day because the light-requiring step in chlorophyll biosynthesis can be activated^45^, we speculated that *CCA1* may enhance heterosis for growth in hybrids in middle of the infection day (6 hpi). In addition, considering that only FCS showed significantly heterosis for defense (Fig. 1a, f, g and Supplementary Fig. 12d) and *CCA1* showed above-high parent expression pattern only in FCS hybrids at 21 hpi that occurred specifically upon pathogen infection (Fig. 1d, e and Supplementary Fig. 2), we speculated that *CCA1* may confer heterosis for defense before the dawn of first infection day (21 hpi), the time that precisely precedes the peak of expression of SA biosynthetic genes and the burst of SA (24 hpi). Thus, *CCA1* may confer growth and defense heterosis at different time points in a day, enabling hybrids to successfully achieve significant defense heterosis without losing their growth vigor.

**Fig. 4:**
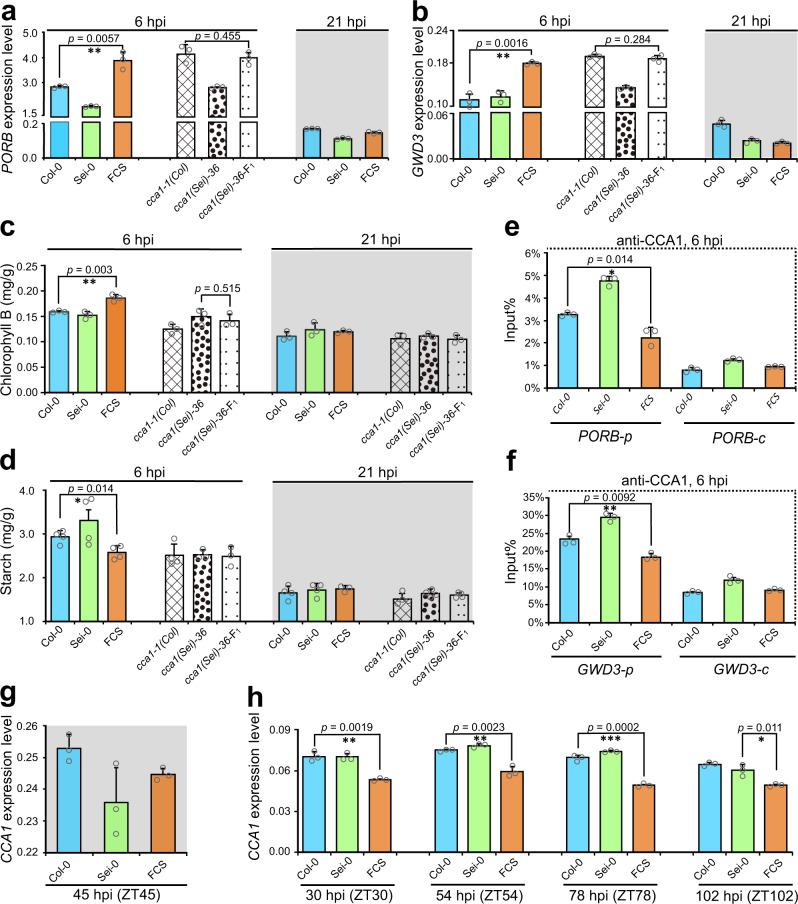
*CCA1* eliminates defense-growth heterosis trade-offs in hybrids by a time-scheduled regulation strategy. qPCR analyses of *PORB*’s (**a**) and *GWD3*’s (**b**) expression level in the F_1_ hybrids and parents of Col-0 × Sei-0 at 6 hpi and 21 hpi and of *cca1-1(Col)* × *cca1(sei)-36* at 6 hpi. Data are standardized for the abundance of the *ACTIN2* transcript. Data are shown as the mean ± SD (*n* = 3, *n* indicates biological replicates). **c** Chlorophyll B content of inoculated leaves in the F_1_ hybrids and parents of Col-0 × Sei-0 and *cca1-1(Col)* × *cca1(sei)-36* at 6 hpi and 21 hpi. Data are shown as the mean ± SD (*n* = 4, *n* indicates biological replicates, six leaves for each biological replicate). **d** Starch content in the inoculated leaves after removing soluble sugar in the F_1_ hybrids and parents of Col-0 × Sei-0 and *cca1-1(Col)* × *cca1(sei)-36* at 6 hpi and 21 hpi. Data are shown as the mean ± SD (*n* = 4, *n* indicates biological replicates, six leaves for each biological replicate). ChIP-qPCR analyses of promoter fragments that contained the “evening element” motif (*PORB-p* and *GWD3-p*) and exon fragments (*PORB-c* and *GWD3-c*) of *PORB* (**e**) and *GWD3* (**f**) in F_1_ hybrids and their parents using an anti-CCA1 antibody at 6 hpi. ChIP values were normalized to their respective DNA inputs. The results are representative of three biological replicates with measurements taken from independent samples grown and processed at different times. Data are shown as the mean ± SD (*n* = 3, *n* indicates biological replicates). qPCR analyses of *CCA1* expression in F_1_ hybrids and the parents of Col-0 × Sei-0 at 45 hpi (ZT45) (**g**) and at 30 hpi (ZT30), 54 hpi (ZT54), 78 hpi (ZT78), and 102 hpi (ZT102) (**h**). The expression level of *CCA1* were shown as the mean ± SD (*n* = 3, *n* indicates biological replicates). Data are standardized for the abundance of the *ACTIN2* transcript. hpi hours post inoculation. *p value < 0.05; **p value < 0.01; and ***p value < 0.001 (two-tailed Student’s *t* test).

Since the evening element-containing above-high parent DEGs in FCS were significantly enriched in starch metabolism and photosynthesis pathway, which tightly correlated with plant growth (Supplementary Fig. 1), we first focused on *PORA* and *PORB*, which encode protochlorophyllide oxidoreductases a and b, and mediate the only light-requiring step in chlorophyll biosynthesis^45^. The upregulation of *PORA* and *PORB* increases the content of chlorophyll a and b, which are essential for photosynthesis in higher plants^46^. We found that *PORB*’s expression level was only significantly upregulated in FCS compared with that in both parents at 6 hpi, but not at 21 hpi (the same time as ZT6 and ZT21 in Fig. 1d), and this above-high parent of *PORB*’s expression pattern in FCS was completely dependent on *CCA1* (Fig. 4a). In contrast to *PORB*, there is no difference between FCS hybrids and their parents for *PORA*’s expression at both 6 hpi and 21 hpi, and no difference between the *CCA1*-mutated hybrids and parents (Supplementary Fig. 14a). Similar to the expression of *PORB*, two typical genes involved in starch degradation, *GWD3* and *DPE1*^47,48^, were also significantly upregulated in FCS only at 6 hpi but not at 21 hpi and not in the *CCA1*-mutated hybrids compared with those in *CCA1*-mutated parents (Fig. 4b and Supplementary Fig. 14b). We also observed that the expression level of *PORB*, *GWD3*, and *DPE1* in the *CCA1*-mutated parents and hybrids were significantly higher than those in the wild-type parents and hybrids, indicating that *CCA1* inhibits these growth-related genes’ expression. The above-high parent expression of *PORB*, *GWD3*, and *DPE1* corresponds to the below-low parent expression of *CCA1* in FCS at 6 hpi (Fig. 1d), which significantly reduced the inhibition of *CCA1* on these growth-related genes in hybrids compare with that in parents.

Consistent with the above-high parent expression patterns of *PORB, GWD3* and *DPE1* in hybrids (Fig. 4a, b and Supplementary Fig. 14b), the content of chlorophyll b in the inoculated leaves of FCS was significantly higher than that in the leaves of both parents at 6 hpi, which was not observed at either 21 hpi or in the *CCA1*-mutated hybrids (Fig. 4c), and the content of starch in the inoculated leaves after the removal of soluble sugar was significantly lower in FCS compared with that in both parents at 6 hpi but not at 21 hpi and in *CCA1*-mutated F_1_ hybrids (Fig. 4d). Altogether, these results indicated that a greater accumulation of chlorophyll and a more rapid degradation of starch occurs specifically at 6 hpi (the middle of the infection day) in hybrids, and the significant increase in the activation of growth-related pathways in the hybrids was totally dependent on *CCA1*.

Since the promoter of these chlorophyll biosynthetic and starch degradative genes contain CCA1 binding motif (evening element), we speculated that *CCA1* contributes to the growth vigor of FCS hybrids at 6 hpi through direct binding to these genes’ promoter to regulate their differential expression between the hybrids and parents. Besides, protein level measurement also shows that CCA1 proteins were differentially expressed between the hybrids and parents at both 6 hpi and 21 hpi (Supplementary Fig. 15). By the ChIP experiments, we found that CCA1 was significantly enriched in the promoter regions that contained the “evening element” but not in the exon region of these starch metabolic and photosynthetic genes (Fig. 4e, f and Supplementary Figs. 14c, 16, and 17), identical to the binding of CCA1 to the *TOC1*’s promoter (Supplementary Fig. 14d) which has been previously reported^49^. These results demonstrate that *CCA1* inhibits the expression of growth-related genes by directly binding to their promoters. Moreover, this enrichment was significantly less in the FCS hybrids than that in both parents for all three of these genes but not for the control gene *ACTIN2* at 6 hpi (Fig. 4e, f and Supplementary Fig. 14c, e), which is consistent with the below-low parent expression pattern of *CCA1* in FCS hybrids at 6 hpi (Fig. 1d). In addition, the enrichment of CCA1 on the promoter of these chlorophyll biosynthetic and starch degradative genes did not differ between the FCS hybrids and parents at both 3 hpi and 24 hpi, which is consistent with the lack of differential expression pattern of *CCA1* between the FCS hybrids and parents at 3 hpi and 24 hpi (Fig. 1d and Supplementary Figs. 16 and 17). Taken together, these results show that below-low parent expression of *CCA1* in hybrids at 6 hpi significantly eliminated the inhibition of *CCA1* on starch metabolic and photosynthetic genes, leading to the above-high parent expression pattern of growth-related genes in hybrids.

In summary, all of these results described above illustrate that when the pathogen invades, the above-high parent expression of *CCA1* at 21 hpi regulates the histone modifications of SA biosynthetic genes and enhances the burst of SA at 24 hpi in the hybrids. At the meanwhile, the below-low parent expression of *CCA1* at 6 hpi specifically leads to the above-high parent expression of growth-related genes and more activation of starch metabolic and photosynthetic pathway in hybrids. With this time-scheduled regulation strategy, a single gene, *CCA1*, successfully confers heterosis for defense in hybrids without influencing their growth vigor, even significantly promotes growth heterosis in hybrids under pathogen invasion.

### *CCA1* rhythmically balances defense and growth heterosis

Subsequently, to illuminate whether *CCA1* enhances defense heterosis of hybrids before dawn and promotes growth heterosis of hybrids in middle of the day in a diurnal manner, we verified the expression level of *CCA1* in FCS hybrids and parents in the middle of day and before dawn of every infection day. We found that *CCA1* returns to the mid-parent expression pattern in FCS hybrids at 24 hpi (Fig. 1d). In addition, no significant difference in *CCA1*’s expression was detected between FCS hybrids and parents at dawn of the second infection day (45 hpi) (Fig. 4g), accompanied by mid-parent level of SA accumulation in the FCS hybrids at 48 hpi (Fig. 2b). Conversely, the below-low parent expression pattern of *CCA1* and significantly more accumulation of chlorophyll was detected in the FCS hybrids at 30, 54, 78, and 102 hpi (the middle of 2–5 infection days) (Fig. 4h and Supplementary Fig. 18). Thus, the inducible above-high parent expression pattern of *CCA1* before dawn specifically occurred on the first day after infection, which not only effectively promotes heterosis for defense in hybrids precisely ahead of SA burst, but also avoids growth consumption caused by constitutive activation of *CCA1* at dawn for the whole infection day. Moreover, combined with rhythmically increasing the growth heterosis in the middle of every infection day, the hybrids successfully achieved remarkable defense heterosis without growth vigor penalties, and even significantly promote growth heterosis.

The core loop of eukaryotic circadian clocks consists of three transcription factors: two partly redundant morning phase factors, CCA1 and LHY, and the evening phase TOC1. *CCA1/LHY* and *TOC1* are repressors of each other’s expression^36,37^. Loss function of any of these core loop genes results in a short period of *CCA1*’s clock but maintains the rhythmic expression of *CCA1*^44,49–53^. Besides, *TOC1*, *CCA1*, and *LHY* can also directly repress expression of the evening complex (EC) genes, such as *EARLY FLOWERING 3* (*ELF3*) and *ELF4*^54^. Mutation of *ELF3* or *ELF4* confers the arrhythmicity of *CCA1*’s expression^50,55–57^. To further explore whether the change in the rhythmic activity of *CCA1* influences the *CCA1*-mediated balanced defense and growth vigor in hybrids, we generated CRISPR/CAS9-based knockout lines of *LHY*, *TOC1*, *ELF3*, and *ELF4* in the Sei-0 background, and each had a significantly decreased level of transcription in the mutated lines compared with that in the wild-type (Supplementary Figs. 6, 7, 21, and 22). By calculating the bacterial number at 5 dpi, the expression level of *PR1* at 1 dpi and the fresh weight of rosette at 5 dpi in the *LHY*-mutated and *TOC1*-mutated hybrids, we found that heterosis for both defense and growth in FCS after *Pst* DC3000 inoculation was not influenced by the mutation of either *LHY* or *TOC1* (Supplementary Figs. 6, 7, 19, and 20). Conversely, the mutation of *ELF3* or *ELF4* significantly decreased heterosis for defense and for growth of FCS (Supplementary Figs. 21 and 22). In addition, the below-low parent expression pattern of *CCA1* at 6 hpi and above-high parent expression pattern of *CCA1* at 21 hpi in FCS hybrids remained in both *LHY* and *TOC1*-mutated hybrids, but was abolished by the mutation of *ELF3* and *ELF4* (Supplementary Fig. 23). These findings demonstrate that both *CCA1* and its rhythmic expression are necessary for defense and growth vigor in hybrids.

### Expression variations of *CCA1* in hybrids correlated with its altered histone modification rhythms

**Fig. 5:**
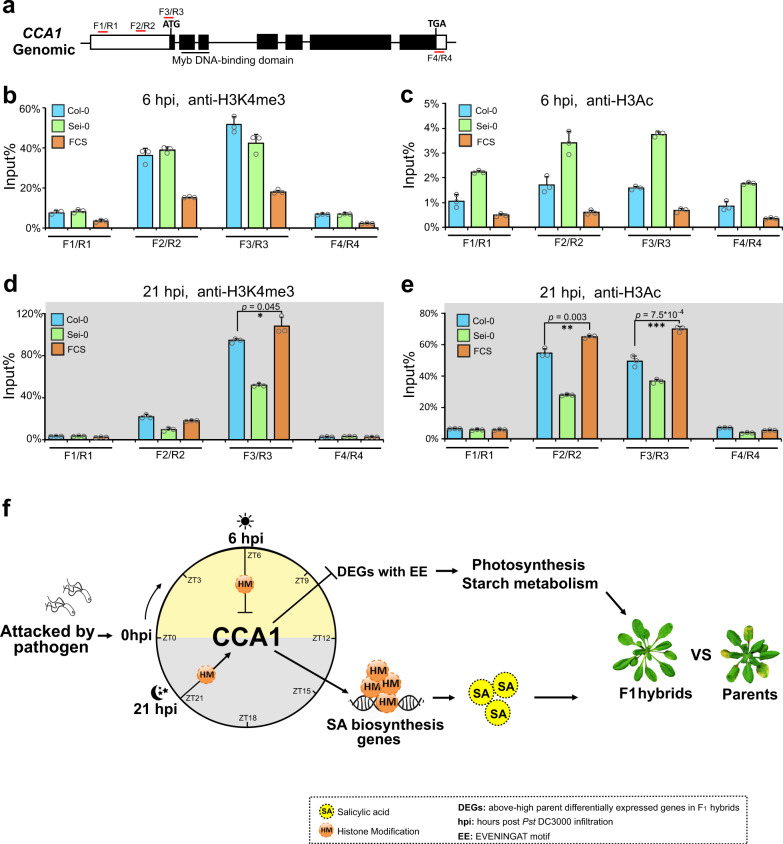
Expression variations of *CCA1* in hybrids correlated with its altered histone modification rhythms. **a** Regions of *CCA1* used for the ChIP-qPCR assays. ChIP-qPCR analyses of four fragments (shown at **a**) of *CCA1* in the wild-type F_1_ hybrids and parents using an anti-H3K4me3 antibody at 6 hpi (**b**) and 21 hpi (**d**). ChIP-qPCR analyses of four fragments (shown at **a**) of *CCA1* in the wild-type F_1_ hybrids and parents using an anti-H3Ac antibody at 6 hpi (**c**) and 21 hpi (**e**). ChIP values in **b**–**e** were normalized to their respective DNA inputs. The results are representative of three biological replicates, with measurements taken from independent samples grown and processed at different times. Data are shown as the mean ± SD (*n* = 3, *n* indicates biological replicates). **f** Working model of how *CCA1* coordinates enhanced heterosis for defense and for biomass in hybrids under pathogen invasion. *p value < 0.05; **p value < 0.01; and ***p value < 0.001 (two-tailed Student’s *t* test).

Previous studies implied a strong correlation between diurnal histone modifications, such as H3 lysine 4 trimethylation (H3K4me3) or acetylated H3 (H3Ac) and the rhythmic expression of *CCA1*^58,59^. To elucidate why *CCA1* exhibited a below-low parent expression pattern in the FCS hybrids at 6 hpi, but conversely, showed an above-high parent expression pattern in FCS hybrids at 21 hpi, we examined the levels of these two modifications on *CCA1* in hybrids and parents at 6 hpi and 21 hpi, respectively. Both H3K4me3 and H3Ac, which mark active transcription^60^, were enriched in regions that surrounded the *CCA1* TSS at 6 hpi and 21 hpi, and the enrichment of these two modifications at 21 hpi was significantly higher than that at 6 hpi (Fig. 5a–e), which was consistent with the higher expression of *CCA1* at 21 hpi than at 6 hpi (Fig. 1d). In addition, it is worth noting that both H3K4me3 and H3Ac displayed significantly lower enrichment at 6 hpi but significantly higher enrichment at 21 hpi in FCS compared with those of both parents (Fig. 5a–e), which is consistent with the below-low parent expression pattern of *CCA1* at 6 hpi and above-high parent expression pattern of *CCA1* at 21 hpi in the FCS hybrids compared with those of the parents (Fig. 1d). Taken together, these results suggest that the altered amplitude of histone modification rhythms of *CCA1* is likely to be the underlying mechanism for the observed significant changes in the transcription of *CCA1* between the hybrids and parents at different time points of day.

In summary, our findings revealed that a single protein, CCA1, confers significant heterosis for defense in hybrids without consuming growth vigor upon pathogen invasion, and we uncovered a novel time-scheduled mechanism for controlling heterosis for different traits. Before the dawn of first infection day, the epigenetic activation of *CCA1* enhances the level of acetylated H3 for the SA biosynthetic genes and promotes a higher burst of SA in the hybrids, leading to significant heterosis for defense. In the middle of every infection day, the diurnal epigenetic repression of *CCA1* leads to the rhythmic upregulation of the downstream genes that contain the “evening element” involved in photosynthesis and starch degradation and then activates chlorophyll synthesis and starch degradation, eliminating defense-growth heterosis trade-offs in hybrids, and even dramatically enhancing the growth vigor in hybrids after pathogen invasion (Fig. 5f). Altogether, *CCA1* coordinates heterosis for both defense and growth by enhancing the disease resistance ability of hybrids at the exact time that precedes the burst of SA to avoid the growth consumption caused by the constitutive activation of *CCA1* at dawn of all infection day; and rhythmically improving the growth vigor of hybrids when the light-requiring step in chlorophyll biosynthesis could be activated and maintaining this growth enhancement in a diurnal manner.

## Discussion

### *CCA1* conferred heterosis for defense beyond the regulation of salicylic acid

In this study, we found that *CCA1* confers heterosis for defense by promoting SA biosynthesis in hybrids, which is tightly associated with the enhanced H3Ac modification of SA biosynthetic genes in hybrids regulated by *CCA1* (Fig. 2). SA is the major phytohormone produced in response to invasion by biotrophic and hemibiotrophic pathogens^35,61^. The production of SA leads to the upregulation of defense pathways, as well as systemic acquired resistance^62^. In this study, we found that the epigenetic activation of *CCA1* before dawn in hybrids specifically occurred on the first day of infection. This precise regulation is not only effective at increasing the accumulation of SA in hybrids but it exactly precedes the burst of SA, thus avoiding the growth consumption caused by the untimely constitutive activation of *CCA1* during all infection days. It also enables hybrids to initiate the strong immune response at the very early stage of pathogen invasion. This early above-high parent accumulation of SA may also improve the systemic acquired resistance of hybrids, which benefits the plants by increasing their resistance to multiple types of pathogen invasion. In addition to SA biosynthetic pathway, several studies have shown that the resistance against *Pst* DC3000 is controlled by the circadian clock at multiple levels, such as the opening of stomata^44,54^, the pattern-triggered immunity (PTI)-induced ROS burst^63^, and the regulation of immune genes induced by MAP kinases^64^. Therefore, it is worth exploring the mechanisms that underlie how *CCA1* contributes to heterosis for defense through the regulation of pathways other than SA biosynthesis. Identifying the target genes of *CCA1* involved in *Pst* DC3000 immunity, functionally characterizing these genes, and exploring their differences in expression between hybrids and parents might be a good starting point.

### Multiple strategies have evolved in hybrids to eliminate defense-growth heterosis trade-offs

In our previous study^10^, we found that hybridization might induce a “primed state” before pathogen invasion, which is characterized by significantly enhanced H3Ac in the promoter regions of SA biosynthetic genes in hybrids. Although this enhanced H3Ac did not induce the expression of these genes ahead of pathogen attack, but it enables a more rapid and stronger activation of SA biosynthesis and defense-related genes in hybrids upon pathogen invasion. This “primed state” effectively reduces the energetic cost for higher defense ability in hybrids.

Taken together, hybrids evolved multiple strategies to reduce the magnitude of defense-growth heterosis trade-offs. Firstly, before pathogen invaded, the hybrids were primed to a physiological “state of readiness,” which prepared them for a more rapid and stronger defense response and effectively reduced the fitness cost for higher defense ability in hybrids once the pathogen invaded. Secondly, upon pathogen attack, hybrids can coordinate the defense and growth heterosis at different time points in a day. This precisely enhances the defense heterosis before dawn when exactly precedes the burst of SA (24 hpi), and promotes the growth heterosis in the middle of the day when the light-requiring step in chlorophyll biosynthesis could be activated. Thirdly, hybrids eliminate the defense-growth heterosis trade-offs by maintaining epigenetic repression of *CCA1* in the middle of every infection day in a diurnal manner but inducing the epigenetic activation of *CCA1* at dawn specifically on the first infection day, which effectively avoids the growth consumption caused by the constitutive activation of *CCA1* at dawn of all infection days. Through these three levels of smart strategies, hybrids not only achieved significantly heterosis for defense without growth vigor costs, but also even dramatically enhance growth heterosis after pathogen infection. It is worth noting that all these strategies are associated with an alteration in the histone modifications in the hybrids. Why hybrids appear to have different histone modifications from their parents is a matter that certainly merits further investigation.

What is more, we noted that the expression level of *CCA1* in FCA only manifested a below-low parent expression pattern at 6 hpi, but there was no differential expression of *CCA1* between FCA and its parents at 21 hpi (Fig. 1e and Supplementary Fig. 2b), which is consistent with the fact that FCA showed only significant heterosis for growth, but no evident heterosis for defense upon pathogen invasion (Figs. 1a and 3d and Supplementary Fig. 12d). In addition, the mutation of *CCA1* only abolished the growth vigor of FCA hybrids, but it did not affect the defense heterosis of FCA hybrids (Fig. 3d, e and Supplementary Fig. 12). Moreover, the differentially expression of *CCA1* between F_1_ hybrids and parents at 21 hpi and 6 hpi contributes to whether the F_1_ hybrids showed heterosis for defense and for growth when pathogen attack, respectively (Supplementary Figs. 9 and 13). All these results implied that *CCA1* can precisely regulate growth heterosis and defense heterosis separately at 6 hpi and 21 hpi, which also benefits the coordination of *CCA1* in defense and growth heterosis in different *Arabidopsis* hybrids.

### Circadian clock regulates heterosis in a special way

Our results in Fig. 3 indicating the important role of *CCA1* in regulating heterosis for growth without pathogen inoculation is consistent with a previous study^6^ that explained the growth vigor in allotetraploids and hybrids. In addition, our study for the first time showed that upon pathogen attack, the circadian clock is also essential for heterosis for defense, and more importantly, is crucial for coordinating the trade-offs between defense and growth heterosis. These results further illustrate the important role of *CCA1* in regulation of heterosis, not only under noninfection growth conditions, but also under pathogen infection.

The circadian clock integrates environmental signals with internal cues to enable proper growth, development, and response to stimuli^65^. A series of studies have shown that the circadian clock is critical for plant innate immunity^54^. We found that the circadian clock regulates heterosis for defense and balances defense-growth heterosis in a singular way, which differs from that of immunity regulation. First, the role of defense of a clock gene is not associated with its specific role in the plant clock. A mutation in any of the clock genes, such as *CCA1, LHY, TOC1, ELF3*, and *ELF4*, conferred enhanced disease susceptibility to *P. syringae* despite the fact that these genes play different roles in clock precision^54^. Conversely, we found that *CCA1* is the specific core loop genes that confers both defense and growth heterosis in hybrids; neither *LHY* nor *TOC1* were involved in this progress (Figs. 1 and 3 and Supplementary Figs. 6, 7, 19, and 20). These observations suggest that although clock genes work together to maintain clock precision, some clock genes could affect the specific output to heterosis for defense. This is the case for *CCA1*, since it is the only circadian core loop oscillator that enables coordinated heterosis for both disease resistance and growth in the same hybrid. Second, in contrast to circadian-regulated immunity, the change in rhythmic activity of *CCA1* caused by a clock mutation does not predict the pathogen resistance of mutants. In the diurnal light condition, small changes in the rhythmic activity of *CCA1*, such as keeping the expression of *CCA1* rhythmical but changing its period of expression, as caused by a *LHY* or *TOC1* mutation, has no influence in heterosis for both defense and growth, because *CCA1* maintains below-low and above-high parent expression patterns in mutated hybrids at 6 and 21 hpi, respectively (Supplementary Figs. 6, 7, 19, 20, and 23). However, if we abolish the rhythmic differential expression pattern of *CCA1* in hybrids, such as by mutating *ELF3* or *ELF4*, which confers arrhythmicity and no differential expression of *CCA1* between hybrids and parents (Supplementary Fig. 23), the growth heterosis was decreased significantly and defense heterosis was abolished in the mutated F_1_ hybrids, although *CCA1* still functions normally in these hybrids (Supplementary Figs. 21–23).

We are still at the beginning of understanding the role that the circadian clock plays in heterosis for defense and balancing the defense-growth heterosis trade-offs. Much research remains to be done to elucidate the underlying molecular mechanisms in clock–heterosis crosstalk, including but not limited to the identification and systematic characterization of individual clock genes in the regulation of heterosis and their deployed mechanisms, and an exploration of how these clock genes orchestrate the integration of temporal information, such as when pathogen invasion, with heterosis.

### Implication of *CCA1*-mediated balanced defense and growth heterosis in crop breeding

To date, the most efficient strategy to prevent disease is to develop varieties with durable and broad-spectrum resistance^22^. The use of resistance (*R*) genes is a cost-effective strategy for the control of disease, but the easy mutation of the pathogen effectors that trigger *R* gene-mediated resistance leads to a quick loss of resistance and a substantial loss in yield^66^. Conversely, resistance controlled by PTI is nonrace specific and more durable because of its decreased selective pressure for pathogens to overcome host resistance^67^.

In this study, we illustrated how *CCA1* confers enhanced PTI in hybrids accompanied by a significant promotion of growth vigor. The N-terminus MYB-DNA binding domain of *CCA1* is highly conserved among eudicots and monocots^68–70^. In monocotyledonous plants, *CCA1* has been isolated from rice^70^ and maize^68^ and exhibits rhythmicity, with peak expression around dawn, which is consistent with the expression patterns of *Arabidopsis CCA1*^68,70^. These observations imply that the functions of *CCA1* in the plant clock system are highly conserved, and our newly discovered strategy about how hybrids coordinate defense and growth vigor in hybrids has substantial potential applications for crop breeding. To achieve this goal, on the one hand, we can look for a promoter that is induced by pathogen, and enhances the expression of *CCA1* at specific time points to avoid deleterious effects on growth, while maintaining the normal circadian rhythm of *CCA1*, to modify the expression of *CCA1* in crop varieties. Alternatively, we can search for parents which have specific *CCA1* alleles, on the chance that their heterozygotic state in hybrids would confer both defense and growth heterosis. Both attempts will help us to develop new crop varieties or hybrids that have strong, durable disease resistance (by PTI) and without yield penalties.

In conclusion, our results provide strong evidence that *CCA1* serves as the essential circadian oscillator to confer significant defense heterosis in hybrids without influencing growth vigor, which was achieved by improving the heterosis for defense and the growth of hybrids at different time points of a day when both resistance and growth are the most effective and have the least fitness cost. In addition, this was achieved and by precisely promoting defense heterosis only before the SA burst but by rhythmically enhancing growth vigor in the middle of all infection days with a diurnal manner. The novel mechanism revealed in this study is a major step forward toward uncovering how higher plants effectively confer significant defense heterosis without growth vigor penalties, and it will be of substantial interest for crop breeding by hybridization.

## Methods

### Plant materials and growth conditions

The *A. thaliana* accessions Aa-0 (N934) and Sei-0 (N1504) were obtained from the Nottingham A. thaliana Stock Centre (Nottingham, UK). All other accessions, Wl-0 (CS76630), Rue3.1-31 (CS76406), Ema-1 (CS76480), Koch-1 (CS76396), WalhaesB4 (CS76408), ICE107 (CS76364), NC-1 (CS76559), Bd-0 (CS76445), Ak-1 (CS76431), ICE91 (CS76362), Ba-1 (CS76441), Ca-0 (CS76459), Altai-5 (CS76433), Bay-0 (CS955), Ber (CS76448), L*er*-1 (CS6928), and Est (CS76485), were obtained from the Arabidopsis Biological Resource Center (Columbus, OH, USA). Crosses were performed by dissecting immature flowers before anther dehiscence and applying pollen to the exposed pistils. The F_1_ hybrid lines were generated by crossing the indicated parental lines. The F_1_ hybrid of *cca1* and *toc1* mutants were generated by crossing the homozygous and cas9-free CRISPR lines in the Sei-0 or Aa-0 background with *cca1-1* and *toc1-101* in the Col-0 background^44,56^, respectively. Plants were grown on Murashige and Skoog plates containing 1% sucrose at 22 °C under white light conditions (100 μmol m^−2^ s^–1^; 16 h light/8 h dark). Plants for pathogen inoculation were grown under 12-h light and 12-h dark conditions in Percival chambers (AR models) where light, temperature, and humidity could be controlled, and leaves from 3-week-old plants were used. All plants were grown at a controlled temperature (22° ± 0.2 °C) with 65% relative humidity. Light provided by Philips Alto II tubes was set at 100 µmol m^−2^ s^−1^ during the day or LL and 0 µmol m^−2^ s^−1^ at nights. Trays of plants were moved to random positions in the growth rooms every 2 days to reduce positional effects.

### Promoter motif analysis and Gene Ontology analysis

DNA sequences from ~1000 bp upstream of the transcription start sites of the above-high parent DEGs (see Supplementary Data 1) between F_1_ hybrid and parents^10^ were extracted and scanned in the PLACE database^71^. Fisher’s exact test was used to calculate the significance of motifs in these promoters compared with the *Arabidopsis* genome. The *p* value was then adjusted by the Benjamini–Hochberg correction method to obtain the *q* value. Gene Ontology results were extracted from the TAIR10 gene annotation, and a functional enrichment analysis was performed at http://bioinfo.cau.edu.cn/agriGO/.

### Constructs and plant transformation

For the egg cell-specific promoter-controlled CRISPR/Cas9^72^, the single guide RNA (sgRNA) sequences were selected with suggestion of CRISPR-PLANT web program (http://crispr.hzau.edu.cn/cgi-bin/CRISPR/CRISPR), and using pCBC-DT1T2 as the template, the sgRNA-U6-26t-U6-29p-sgRNA cassette was amplified by PCR and cloned into pHEE401. The primers used for plasmid construction and for mutant screening are listed in Supplementary Table 4. The plasmids were transformed into plants using *Agrobacterium* GV3101 and the floral dipping method. Transformants were selected on MS medium containing hygromycin.

### Western blot analysis

Twelve-day-old seedlings grown in Murashige and Skoog plates that contained 1% sucrose at 22 °C under diurnal conditions (12 h light/12 h dark) were collected at 9:00 a.m. (ZT0) for Col-0, Sei-0, Aa-0, and the corresponding *CCA1*-mutated lines. The CCA1 protein was detected on a 10% SDS-PAGE using a rabbit polyclonal antibody against CCA1. The CCA1-specific antibody was generated by ABclonal Biotechnology Co., Ltd. (Wuhan, China) and was used at 1:1000 dilution for western blots. The ImageJ v1.8.0_172 (https://imagej.nih.gov/ij/) was used to quantify protein band intensities.

### RNA extraction and qRT-PCR

Leaves from 3-week-old plants were infiltrated with *Pst* DC3000 (2 × 10^5^ cfu ml^–1^) or a control and collected at different time points. At least five leaves from different plants were pooled in each sample for qRT-PCR. Leaves or 12-day-old seedlings were ground to a powder in liquid nitrogen, and total RNA was extracted using an RNeasy Plant Mini Kit (Qiagen) with an On-Column DNase I digestion treatment. Spectrophotometric and gel electrophoretic analyses were performed to detect the quality of RNA. To synthesize cDNA, 2 µg of RNA was used in the SuperScript III First-Strand Synthesis System (Invitrogen). RT-qPCR analysis (56 °C, 45 s and 45 cycles) was performed using SYBR Premix Ex Taq II mix (Takara) on an ABI7500 real-time PCR detection system (Applied Biosystems). Each experiment was repeated with three independent samples, and qRT-PCR reactions were performed in three technical replicates for each sample. The level of expression was calculated as 2^ΔΔCT^ and then normalized to that of *A. thaliana ACTIN*. All the primers used are listed in Supplementary Table 4.

### Bacterial inoculation and the determination of bacterial growth

*Pst* DC3000 was grown at 28 °C in King’s B medium (10 mg ml^–1^ protease peptone, 1.5 mg ml^–1^ K_2_HPO_4_, 15 mg ml^–1^ glycerol)^73^ supplemented with 25 μg ml^–1^ rifampicin. Mature, fully expanded leaves of 3-week-old plants were infected with suspensions of bacterial cells in 10 mM MgCl_2_ by pressing a 1-ml syringe (without a needle) against the abaxial side of the leaves and forcing the suspension through the stomata into the intercellular spaces. The bacterial dose was 2 × 10^5^ cfu cm^–2^ leaf area (equivalent to OD_600_ = 0.0004). Five days after inoculation, the degree of bacterial growth in plant leaves was determined by harvesting 24–32 infected leaves per sample (approximately eight plants, divided into six to eight replicates with four leaves each), and the weight of each replicate was calculated. Leaves were placed into a microcentrifuge tube containing 1 ml of 10 mM MgCl_2_ and ground with a plastic pestle. This material was diluted, and 45-µl samples were spread on King’s B plates containing 25 μg ml^–1^ rifampicin. The plates were incubated for 2 days at 28 °C. Six to eight replicate samples per genotype were assayed to obtain means and standard deviations, which were determined from the logarithm of the number of cfu per g^2^.

### Determination of endogenous levels of SA

Mature leaves of 3-week-old plants were infected with *Pst* DC3000 at a dose of 2 × 10^5^ cfu cm^–2^ leaf area (equivalent to OD_600_ = 0.0004). At 0, 24, 48, and 72 hpi, samples were collected (~0.2 g of tissue per sample, from approximately six leaves from six plants). Samples were ground in liquid nitrogen, and ~200-mg samples were mixed with 1 ml of ethyl acetate spiked with 200 ng of D_4_-SA used as an internal standard for SA. Phytohormone extraction and quantification were performed with an HPLC-MS/MS (LCMS-8040, Shimadzu, Tokyo, Japan) system. Three replicates were collected for each data point. Statistical analyses were performed using Student’s *t* test of the differences between the two means.

### Chromatin immunoprecipitation

Approximately 2 g of materials was cross-linked with 1% formaldehyde in a vacuum for 35 min and were then ground to powder in liquid nitrogen. The chromatin complexes were isolated, sonicated, and then incubated with anti-AcH3 (Upstate; 06-599, 1:100 dilution), anti-H3K4me3 (Abcam; ab8580, 1:100 dilution), and anti-CCA1 (generated by ABclonal Biotechnology Co., Ltd. (Wuhan, China), rabbit polyclonal antibody, 1:100 dilution) antibodies, which were used in a 10-μl volume for immunoprecipitation. An equal amount of sample without antibody was used as a mock control. The precipitated DNA was recovered and analyzed by qPCR using specific primers listed in Supplementary Table 4. Each ChIP value was normalized to its respective input DNA value. All ChIP-qPCR experiments were independently performed in triplicate.

### Chlorophyll and starch contents

Mature leaves of ~3-week-old plants were infected with *Pst* DC3000 and were then ground to powder in liquid nitrogen. After that, chlorophyll was extracted in the dark with 1 ml of ethanol (95%) at 4 °C for 24 h. The precipitate was washed three to five times with 95% ethanol until it was completely white. The supernatant was transferred to a new tube, and the content of chlorophyll was calculated using spectrophotometric absorbance (*A*) at light wavelengths of 665 and 649 nm, with 95% ethanol as a control^74^. The chlorophyll content is shown as milligrams of chlorophyll per gram of freshly infected leaves

Chlorophyll a (mg g^−1^) = 13.95 × A_665_ − 6.88 × A_649_

Chlorophyll b (mg g^−1^) = 24.96 × A_649_ − 7.32 × A_665._

The content of starch was measured from leaves inoculated with *Pst* DC3000 (~150 mg of fresh weight). The leaves were ground to powder in liquid nitrogen and incubated at 80 °C for 30 min in 80% ethanol to separate the soluble sugar from the starch. The total starch in each sample was then quantified using a kit from Solarbio (BC0700, China) according to the manual.

### Fresh/dry weight measurement

Twenty-day-old soil-grown F_1_ hybrids and parents were infected with *Pst* DC3000 at a dose of 2 × 10^5^ cfu cm^–2^ leaf area (equivalent to OD_600_ = 0.0004). Four leaves were inoculated for each plant. Five days after infiltration, the whole rosette or the infected leaves were weighed. Thirty plants and thirty replicates (four leaves for each replicate) were measured for the whole rosette and for the infected leaves, respectively. A total of 15 or 20 plants were measured for the whole rosette in noninfiltration condition. The dry weight was measured 15 days after incubation at 65 °C for the whole rosette (three plants for each replicate).

### Statistics and reproducibility

Microsoft Excel 2019 was used to analyze qRT-PCR results and determine statistical significance based on the two-tailed Student’s *t* test. Statistical significance was considered when *p* < 0.05. The *n* value corresponds to the number of samples for each column, where the type of sample is indicated in the figure legends. Each experiment was repeated three times independently with similar results.

### Reporting summary

Further information on research design is available in the Nature Research Reporting Summary linked to this article.

## Supplementary information

Supplementary Information

Descriptions of Additional Supplementary Files

Supplementary Data 1

Reporting Summary

## Data Availability

All data that support the findings of this study are provided as a Source Data file and are available from the corresponding authors upon a reasonable request. There are no restrictions on data availability. Source data are provided with this paper.

## Source data

Source Data
