# Supplementary material for: A central circadian oscillator confers defense heterosis in hybrids without growth vigor costs
Source: Nat Commun. 2021 Apr 19;12:2317. doi: 10.1038/s41467-021-22268-z (PMC8055661; doi:10.1038/s41467-021-22268-z)
Supplement: Supplementary file 1 — Supplementary Information [file 41467_2021_22268_MOESM1_ESM.pdf]

Supplementary Figures:

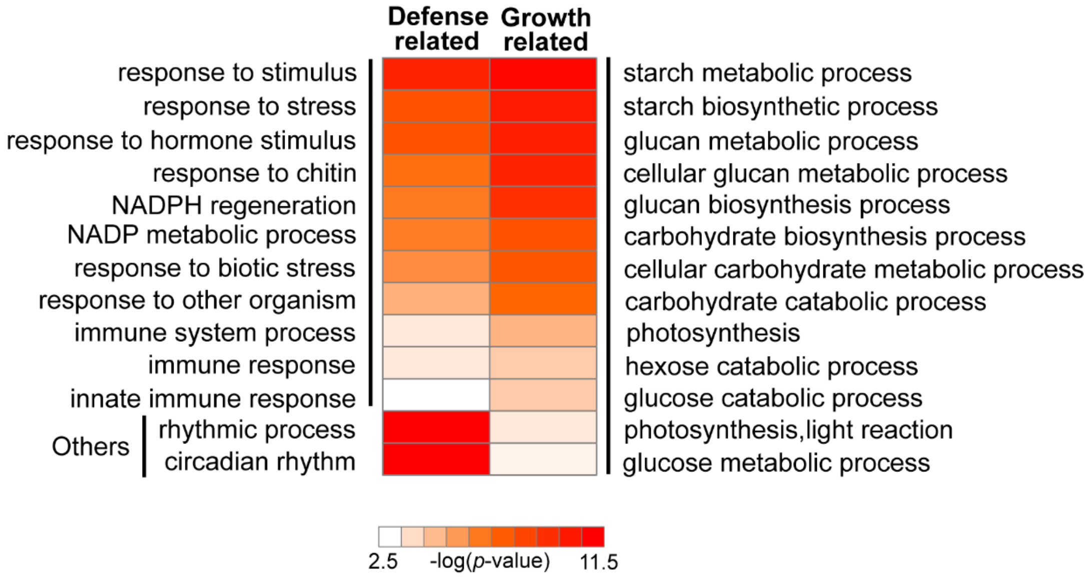

Supplementary Fig. 1. 'evening element'- containing above-high parent DEGs are involved in defence- and growth-related or circadian rhythm pathways.

Selected Gene Ontology (GO) categories for 'evening element'- containing DEGs with 'above-high parent' expression pattern in the F_1 hybrids at 1-, 2-, and 3-days post infiltration with *Pst* DC3000. These DEGs are involved in defence- and growth-related pathways or the circadian rhythm pathway. DEGs, differentially expressed genes.

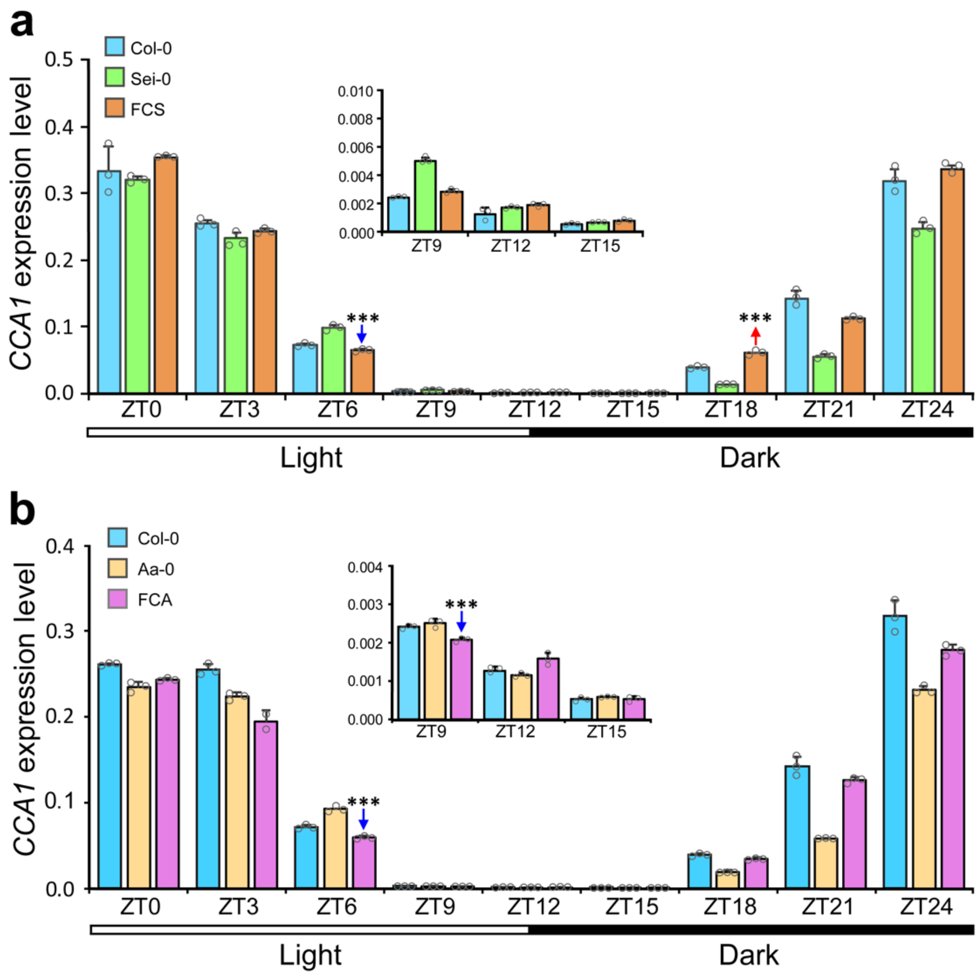

Supplementary Fig. 2. Above-high parent expression of *CCA1* in hybrids at ZT21 did not occurred without pathogen invasion.

a, **b** Quantitative RT-PCR analysis of *CCA1*'s expression level in F_1 hybrids and parents of Col-0 × Sei-0 (**a**) and Col-0 × Aa-0 (**b**) (*ACTIN2* as a control) in a 24-h period (12-h light/12-h dark cycles) starting from dawn (ZT0, 9:00) without infiltration of *Pst* DC3000. Data are shown as the mean ± SD (n = 3, n indicates biological replicates). Arrows indicate significant up-(red) and down-(blue) regulation of *CCA1* in the F_1 hybrids compared to mid-parent value. The *p*-values in **a** are 6.2E-04 for ZT6 and 9.1E-05 for ZT18. The *p*-values in **b** are 3.13E-04 for ZT6 and 5.0E-04 for ZT9. The results are representative of three independent experiments, with measurements that were taken from independent samples grown and processed at different times. '***' *p* value < 0.001 (two-tailed Student's *t test*).

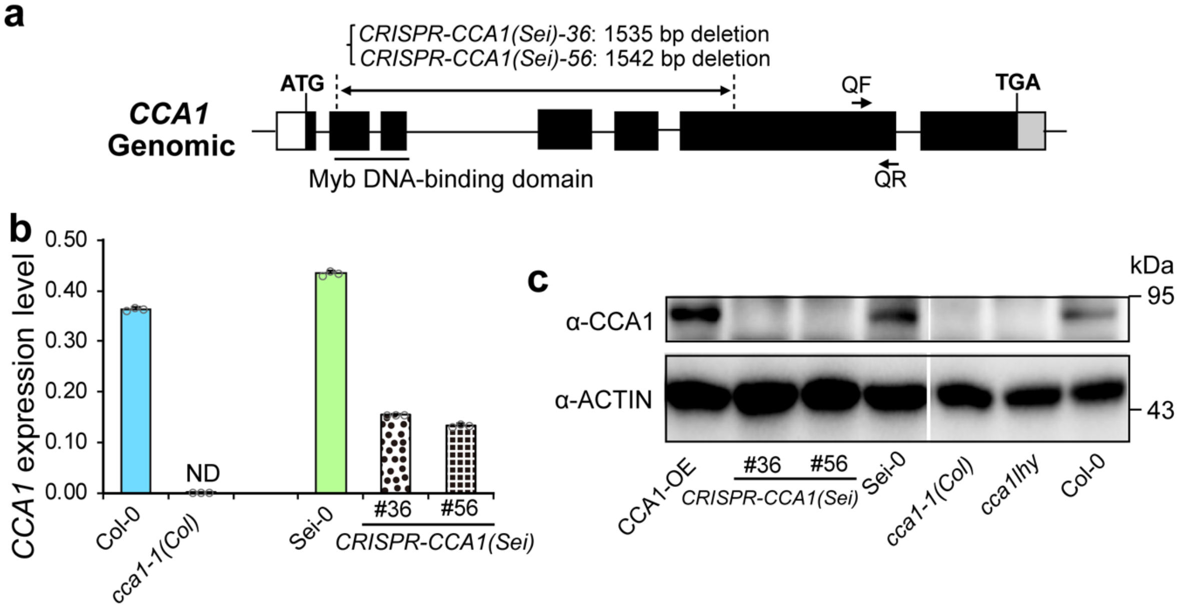

Supplementary Fig. 3. Gene model and transcript/translation level of *CCA1* mutants.

a Gene model of *CCA1-CRISPR* mutants in the Sei-0 background. White box, promoter; black boxes, exons; gray boxes, 3'UTR. Lines with arrowheads indicate the fragment deleted in *CRISPR* mutants. Primers used to test the transcript level of *CCA1* are indicated by arrows. **b**, **c** The transcript (**b**) and translation (**c**) level of *CCA1-CRISPR* mutants and the wild-type at ZT0. The transcript level of *CCA1* (ZT0, 12-day-old seedlings) is shown as the mean ± SD (n = 3, n indicates biological replicates) and is standardized for the abundance of the *ACTIN2* transcript.

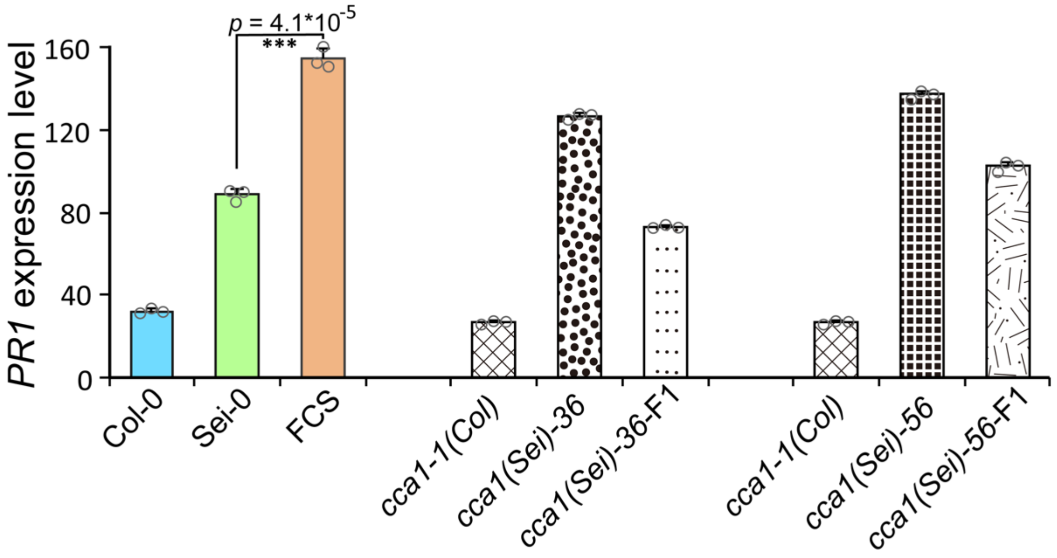

Supplementary Fig. 4. The expression level of *PR1* in wild-type and *CCA1*-mutated hybrids and parents.

qPCR analyses of *PR1*'s expression level of the wild-type and *CCA1*-mutated F_1 hybrids and parents at 24 hours post infiltration with *Pst* DC3000. The expression level of *PR1* was shown as the mean ± SD (n = 3, n indicates biological replicates). Data are standardized for the abundance of the *ACTIN2* transcript. '***' p value < 0.001 (two-tailed Student's *t test*).

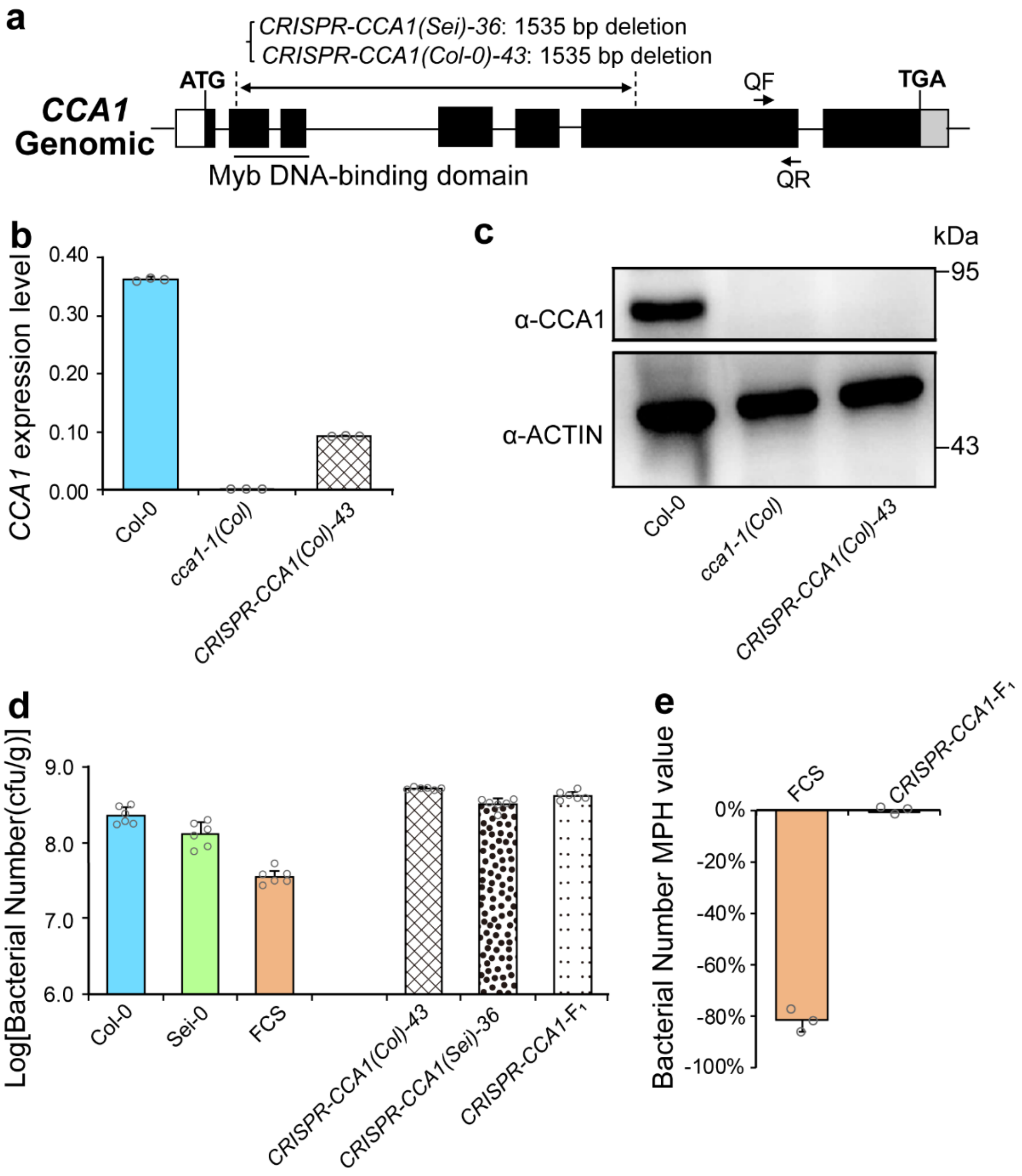

Supplementary Fig. 5. ***CCA1*** **is the key regulator of heterosis for defence.**

a Gene model of *CCA1*-*CRISPR* mutants in Col-0 and Sei-0 background. White box, promoter; black boxes, exons; gray boxes, 3'UTR. Lines with arrowheads indicate the fragment deleted in *CRISPR* mutants. Primers used to test the transcript level of *CCA1* are indicated by arrows. **b**, **c** The transcript (**b**) and translation (**c**) level of *CCA1* mutants and Col-0 at ZT0. The transcript level of *CCA1* (ZT0, 12-day-old seedlings) is shown as the mean ± SD (n = 3, n indicates biological replicates) and standardized for the abundance of the *ACTIN2* transcript. **d** Bacterial titer (log10) at 5 days post infiltration with *Pst* DC3000 for the wild-type and *CCA1*-mutated F_1 hybrids and parents. Bacterial

growth is expressed as the mean value of viable bacteria per gram of leaf tissue ± SD (n = 6, n indicates biological replicates). **e** Mid-parent heterosis (MPH) value calculated by bacterial number at 5 days post infiltration with *Pst* DC3000 for wild-type and *CCA1*-mutated F_1 hybrids. Data are shown as the mean ± SD. The results in **d** and **e** are representative of three independent experiments, with measurements that were taken from independent samples grown and processed at different times.

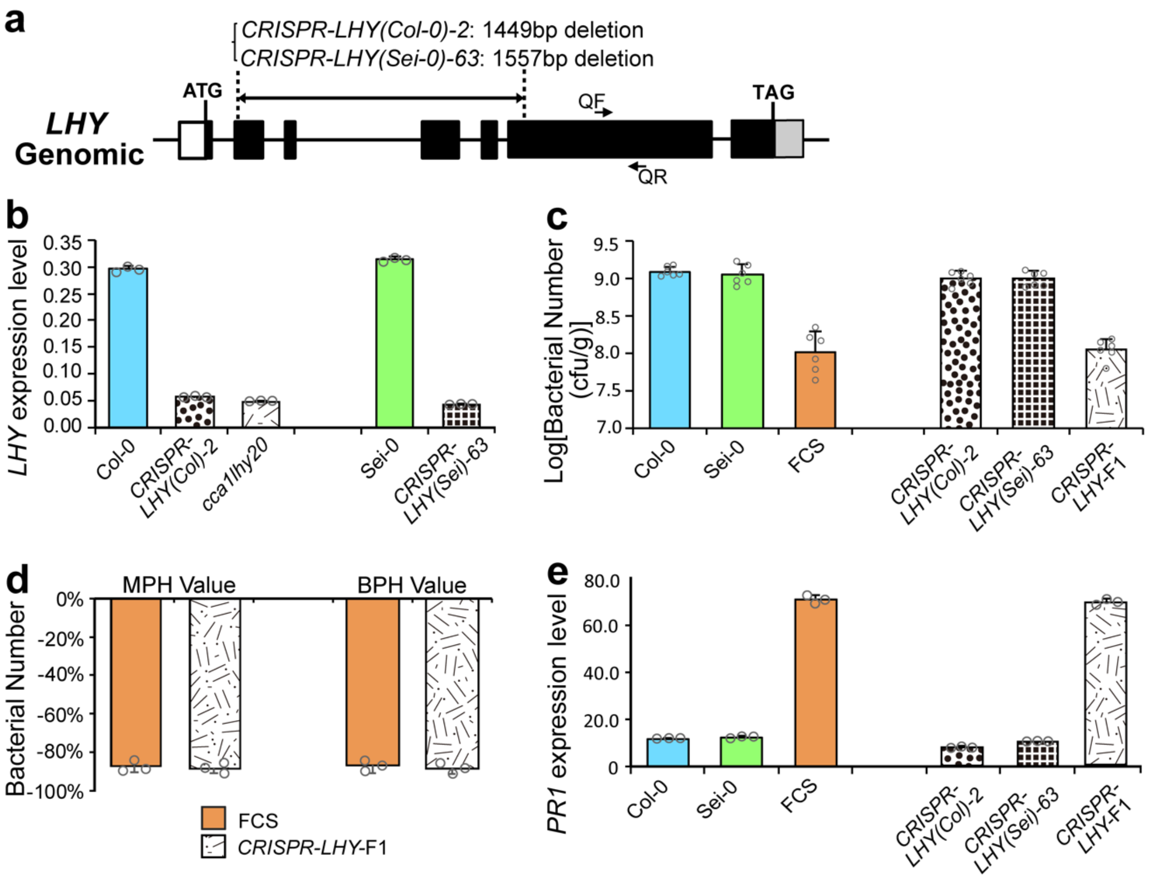

Supplementary Fig. 6. Mutation of *LHY* did not influence the defence heterosis of FCS hybrids.

a Gene model of *LHY-CRISPR* mutants in Col-0 and Sei-0 background. White box, promoter; black boxes, exons; gray boxes, 3'UTR. Lines with arrowheads indicate the fragment deleted in *CRISPR* mutants. Primers used to test the transcript level of *LHY* are indicated by arrows. **b** The transcript level of *LHY* mutants and the wild-type. The transcript level of *LHY* (ZT0, 12-day-old seedlings) is shown as the mean ± SD (n = 3, n indicates biological replicates) and standardized for the abundance of the *ACTIN2* transcript. **c** Bacterial titer (log10) at 5 days post infiltration with *Pst* DC3000 in the wild-type and *LHY*-mutated F_1 hybrids and parents. Bacterial growth is expressed as the mean values of viable bacteria per gram of leaf tissue ± SD (n = 6, n means biological replicates). **d** Mid-parent heterosis (MPH) value and Best-parent heterosis (BPH) value calculated by bacterial number at 5 days post infiltration with *Pst* DC3000 for the wild-type and *LHY*-mutated F_1 hybrids. Data are shown as the mean ± SD. **e** qPCR analyses

of *PR1*'s expression level of wild-type and *LHY*-mutated F_1 hybrids and parents at 24 hours post infiltration with *Pst* DC3000. The expression level of *PR1* is shown as the mean ± SD (n = 3, n indicates biological replicates). Data are standardized for the abundance of the *ACTIN2* transcript. The results in **c** to **e** are representative of three independent experiments, with measurements that were taken from independent samples grown and processed at different times.

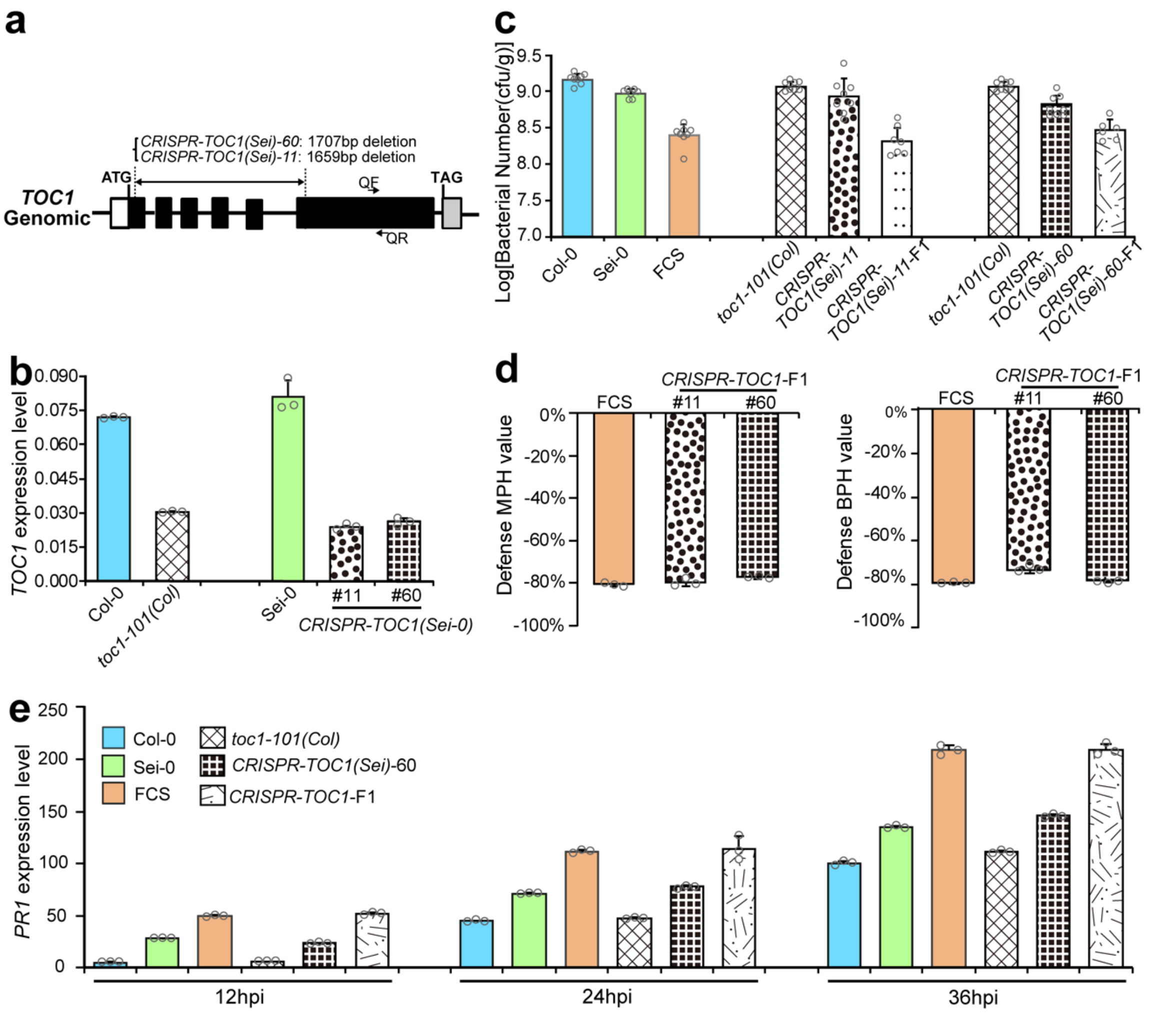

Supplementary Fig. 7. Mutation of *TOC1* did not influence defence heterosis of FCS hybrids.

a Gene model of *TOC1-CRISPR* mutants in the Sei-0 background. White box, promoter; black boxes, exons; gray boxes, 3'UTR. Lines with arrowheads indicate the fragment deleted in *CRISPR* mutants. Primers used to test the transcript level of *TOC1* are indicated by arrows. **b** Transcript level of *TOC1* mutants and the wild-type at ZT12. The transcript level of *TOC1* (ZT12, 12-day-old seedlings) is shown as the mean ±SD (n = 3, n indicates biological replicates) and standardized for the abundance of the *ACTIN2* transcript. **c** Bacterial titer (log10) of the wild-type and *TOC1*-mutated F_1 hybrids and parents at 5 days post infiltration with *Pst* DC3000. Bacterial growth is expressed as the mean values of viable bacteria per gram of leaf tissue ±SD (n = 6, n indicates biological replicates). **d** Mid-parent heterosis (MPH) and Best-parent heterosis (BPH) values

calculated by bacterial number at 5 days post infiltration with *Pst* DC3000 for the wild-type and *TOC1*-mutated F_1 hybrids. Data are shown as the mean ± SD. **e** qPCR analyses of *PR1*'s expression level of the wild-type and *TOC1*-mutated F_1 hybrids and parents at 12, 24 and 36 hours post infiltration with *Pst* DC3000. The expression level of *PR1* was shown as the mean ± SD (n = 3, n indicates biological replicates). Data are standardized for the abundance of the *ACTIN2* transcript. The results in **c** to **e** are representative of three independent experiments, with measurements that were taken from independent samples grown and processed at different times.

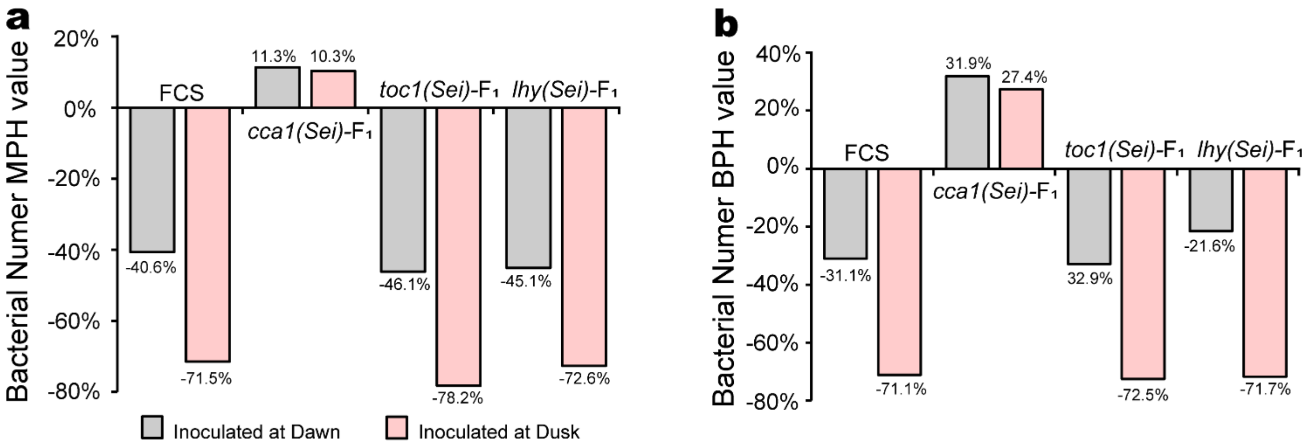

Supplementary Fig. 8. Mutation of *LHY* and *TOC1* didn't influence the increased degree of defence heterosis when inoculated at 'dusk' compared with that at 'dawn'.

Mid-parent heterosis (MPH) (**a**) and Best-parent heterosis (BPH) values (**b**) of the wild-type and *CCA1*, *LHY*, and *TOC1*-mutated F_1 hybrids which were calculated by bacterial number at 5 days post infiltration at dawn and at dusk, respectively. Because the bacterial number at 5 days post infiltration in FCS was significantly less than that in its parents, both the MPH and BPH value of FCS represented by the bacterial number have a negative value in the figure.

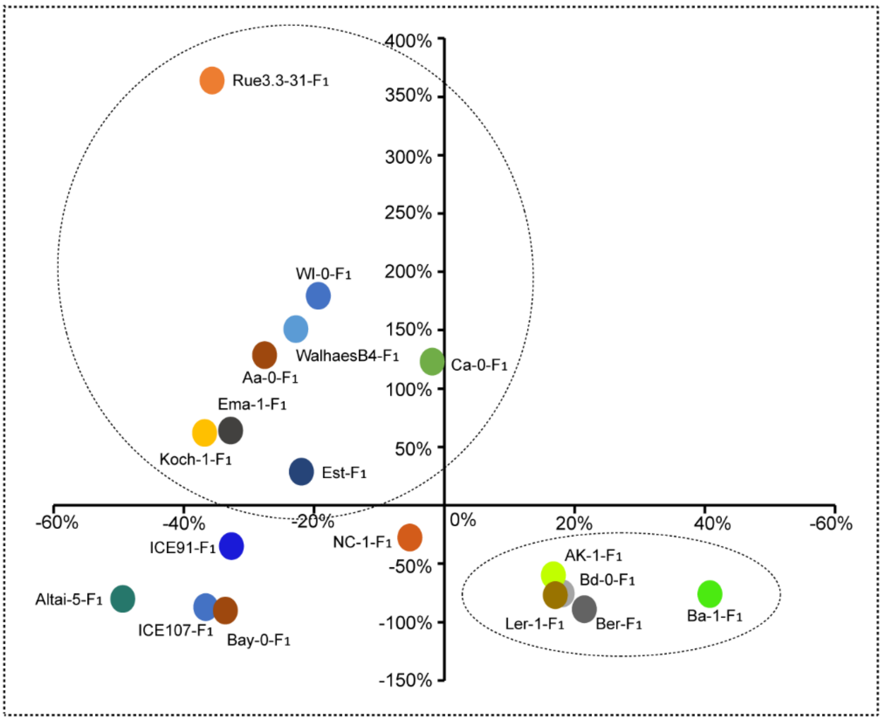

Supplementary Fig. 9. *CCA1* is a common regulator that contributes to heterosis for defence in different *Arabidopsis* hybrids.

Mid-parent heterosis (MPH) value calculated by the bacterial number at 5 days post infiltration (dpi) with *Pst* DC3000 and *CCA1*'s expression level at 21 hours post infiltration (hpi) with *Pst* DC3000 in 18 F_1 hybrids. The 13 F_1 hybrids (72.2%) that are circled show increased or decreased expression of *CCA1* at 21 hpi compared with the mid-parent value (MPV), and, respectively, show heterosis or no heterosis for defence at 5 dpi.

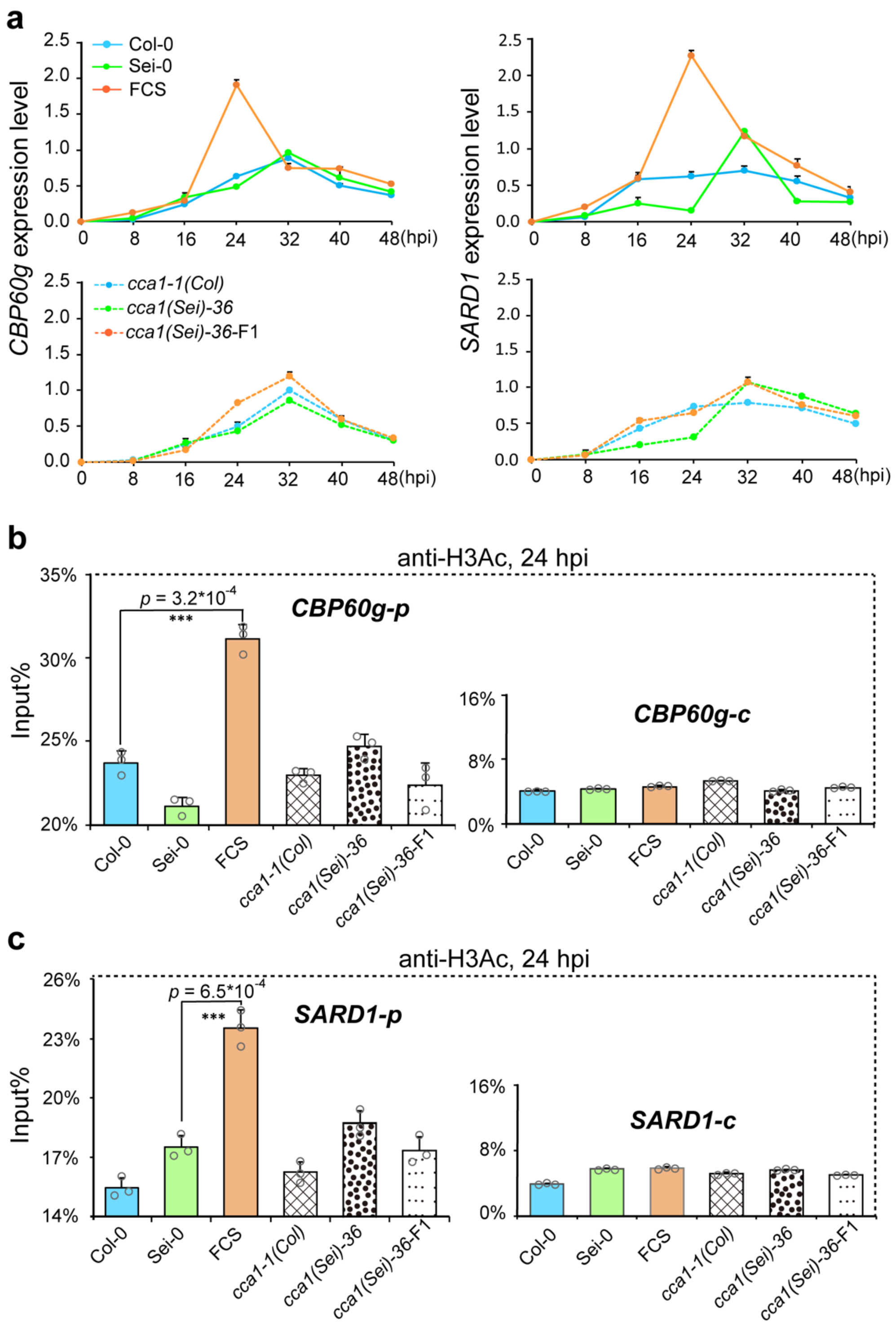

Supplementary Fig. 10. *CCA1* controls the above-high parent expression of *SARD1* and *CBP60g* in hybrids through up-regulation of their H3 acetylation.

a qPCR analyses of *SARD1*'s and *CBP60g*'s expression level in F_1 hybrids and the parents of Col-0 × Sei-0 and *cca1-1* × *cca1(Sei)-36* every 8 hours post-infiltration up to 48 hours. The expression level of *SARD1* and *CBP60g* are shown as the mean ± SD (n = 3, n indicates biological replicates). Data are standardized for the abundance of the

ACTIN2 transcript. hpi: hours post infiltration with *Pst* DC3000. **b**, **c** ChIP-qPCR analyses of promoter fragments (*SARD1-p* and *CBP60g-p*) and exon fragments (*SARD1-c* and *CBP60g-c*) of *SARD1* (**b**) and *CBP60g* (**c**) in wild-type F_1 hybrids and their parents using an anti-H3Ac antibody at 24 hours post infiltration with *Pst* DC3000. ChIP values were normalized to their respective DNA inputs. The results are representative of three independent experiments, with measurements taken from independent samples grown and processed at different times. Data are shown as the mean ± SD (n = 3, n indicates biological replicates). '***' *p* value < 0.001 (two-tailed Student's *t test*).

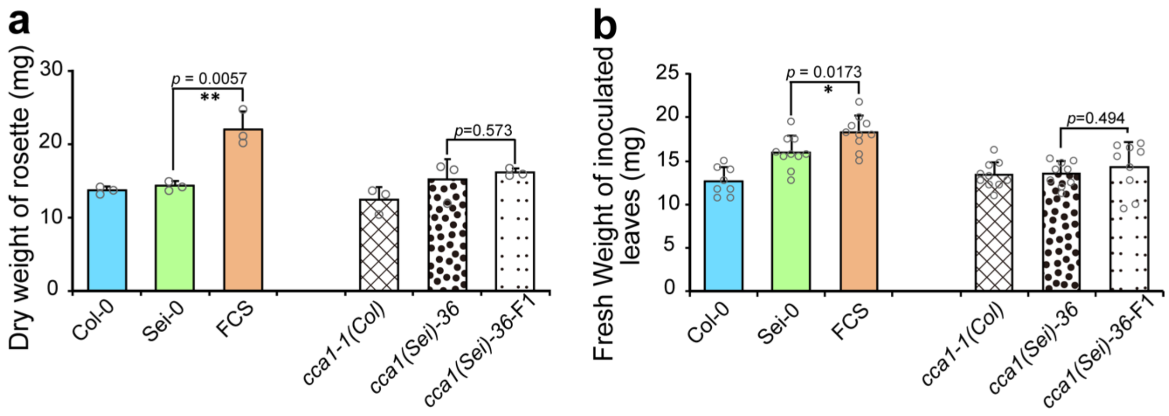

Supplementary Fig. 11. Hybrids achieved significant defence heterosis without consuming growth vigor is completely dependent on *CCA1*.

a Dry weight of the whole rosette of the wild-type and *CCA1*-mutated F_1 hybrids and parents at 5 days post infiltration with *Pst* DC3000. Data are shown as the mean ± SD (n = 30 plants for each genotype). **b** Fresh weight of inoculated leaves of the wild-type and *CCA1*- mutated F_1 hybrids and parents at 5 days post infiltration with *Pst* DC3000. Data are shown as the mean ± SD (n = 6, n indicates biological replicates, 5 leaves for each biological replicate). '*' *p* value < 0.05 and '**' *p* value < 0.01 (two-tailed Student's *t test*).

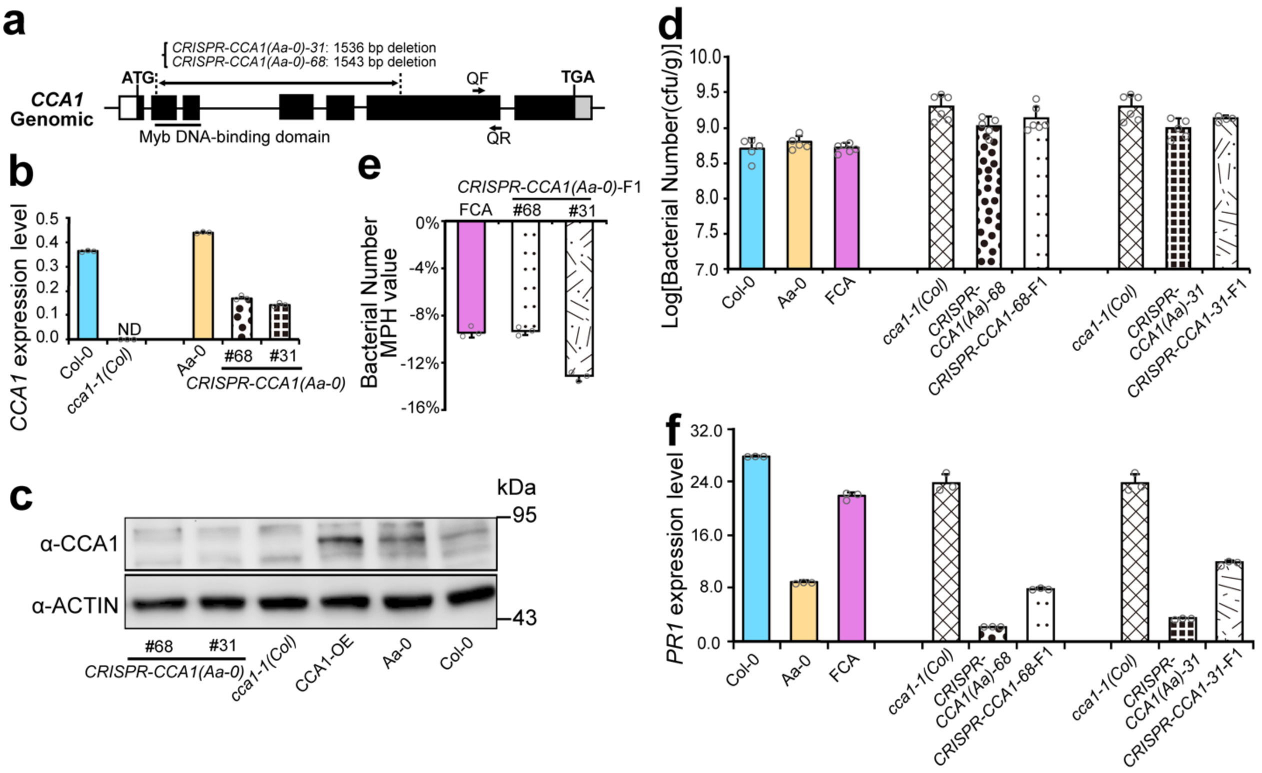

Supplementary Fig. 12. Mutation of *CCA1* results in a lack of heterosis for defence in FCA hybrids.

a Gene model of the *CCA1-CRISPR* mutants in the Aa-0 background. White box, promoter; black boxes, exons; gray boxes, 3'UTR. Lines with arrowheads indicate the fragment deleted in *CRIPSR* mutants. Primers used to test the transcript level of *CCA1* are indicated by arrows. **b**, **c** The transcript (**b**) and translation (**c**) levels of *CCA1* mutants and wild-type. The expression level of *CCA1* (ZT0, 12-day-old seedlings) is shown as the mean ± SD (n = 3, n indicates biological replicates). Data are standardized for the abundance of the *ACTIN2* transcript. **d** Bacterial titer (log10) of the wild-type and *CCA1*-mutated F_1 hybrids and parents at 5 days post infiltration with *Pst* DC3000. Bacterial growth is expressed as the mean values of viable bacteria per gram of leaf tissue ± SD (n = 6, n indicates biological replicates). **e** Mid-parent heterosis (MPH) value calculated by bacterial number at 5 days post infiltration with *Pst* DC3000 for wild-type and *CCA1*-mutated F_1 hybrids. Data are shown as the mean ± SD. **f** qPCR analyses of *PR1*'s expression level of wild-type and *CCA1*-mutated F_1 hybrids and parents at 24 hours post infiltration with *Pst* DC3000. The expression level of *PR1* was

shown as the mean ± SD (n = 3, n indicates biological replicates). Data are standardized for the abundance of the *ACTIN2* transcript. The results in **d-f** are representative of three replicates, with measurements taken from independent samples grown and processed at different times.

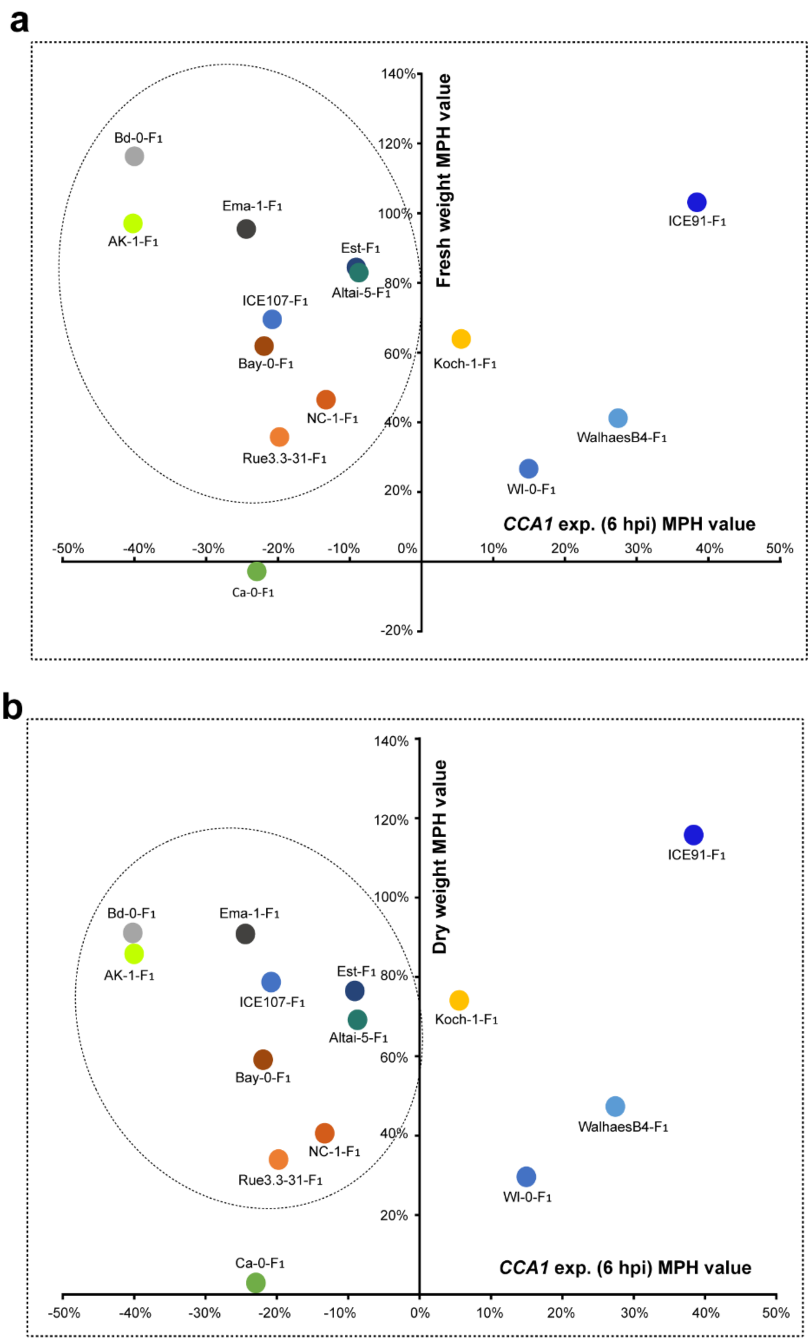

Supplementary Fig. 13. ***CCA1*** **is a common regulator that contributes to heterosis for growth in different** ***Arabidopsis thaliana*** **hybrids.**

Mid-parent heterosis (MPH) value calculated by the rosette fresh weight (**a**) or dry weight (**b**) at 5 days post infiltration (dpi) with *Pst* DC3000 and the *CCA1*'s expression level at 6 hours post infiltration (hpi) with *Pst* DC3000 in 14 F_1 hybrids. Nine F_1 hybrids (64.3%) that are circled show decreased expression of *CCA1* compared with the mid-parent value (MPV) at 6 hpi show heterosis for growth at 5 dpi.

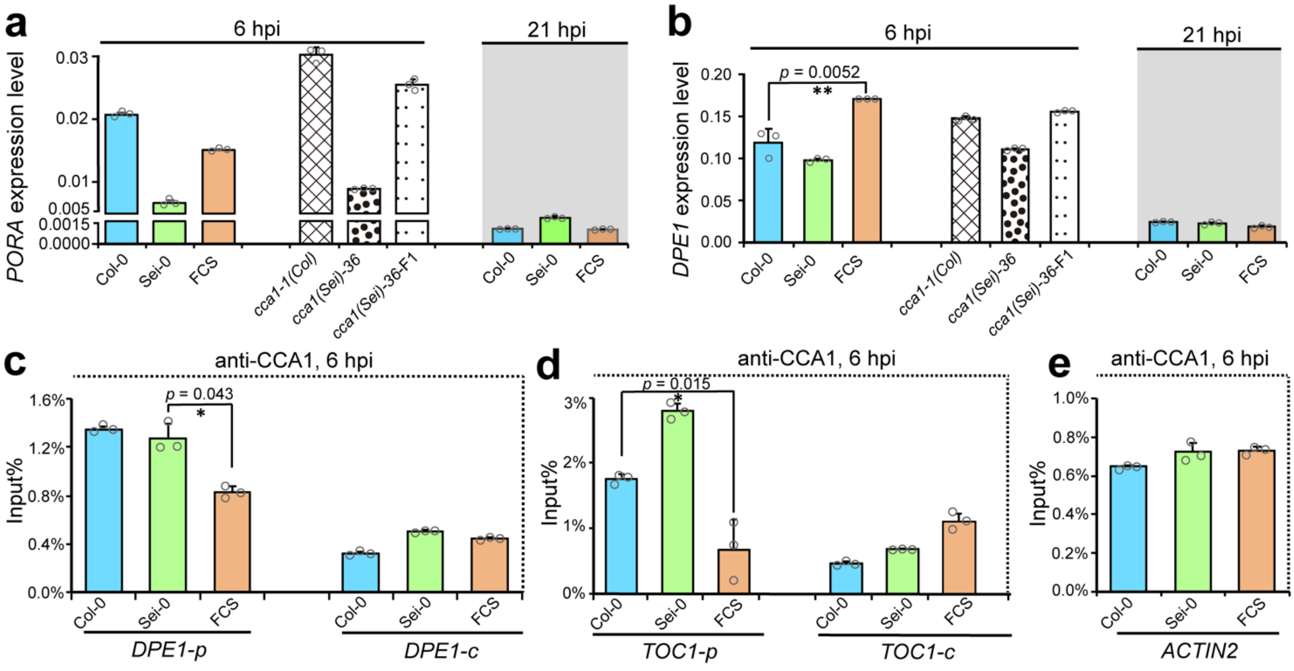

Supplementary Fig. 14. *CCA1* binds to growth-related genes and regulates their differential expression between hybrids and parents after pathogen invasion.

a, **b** qPCR analyses of the expression level of *PORA* and *DPE1* in F_1 hybrids and the parents of Col-0 × Sei-0 at 6 and 21 hpi with *Pst* DC3000 and of *cca1-1(Col)* × *cca1(sei)-36* at 6 hpi with *Pst* DC3000. The expression level of *PORA* (**a**) and *DPE1* (**b**) were shown as the mean ± SD (n = 3, n indicates biological replicates). Data are standardized for the abundance of the *ACTIN2* transcript. **c**, **d** ChIP-qPCR analyses of promoter fragments that contained the 'evening element' motif (*TOC1-p* and *DPE1-p*) and exon fragments (*TOC1-c* and *DPE1-c*) of *TOC1* (**c**) and *DPE1* (**d**) in F_1 hybrids and their parents using the CCA1 antibody at 6 hpi with *Pst* DC3000. *TOC1* was selected as a positive control, indicating that there were no problems with either the *CCA1* antibody or the ChIP system. **e** ChIP-qPCR analyses of *ACTIN2* in F_1 hybrids and their parents using the CCA1 antibody at 6 hpi with *Pst* DC3000. *ACTIN2* was selected as a negative control. ChIP values in **c**, **d**, **e** were normalized to their respective DNA inputs. The results are representative of three independent experiments, with measurements taken from independent samples grown and processed at different times. Data are shown as the mean ± SD (n = 3, n indicates biological replicates). hpi: hours post infiltration. '*' *p* value < 0.05 and '**' *p* value < 0.01 (two-tailed Student's *t test*).

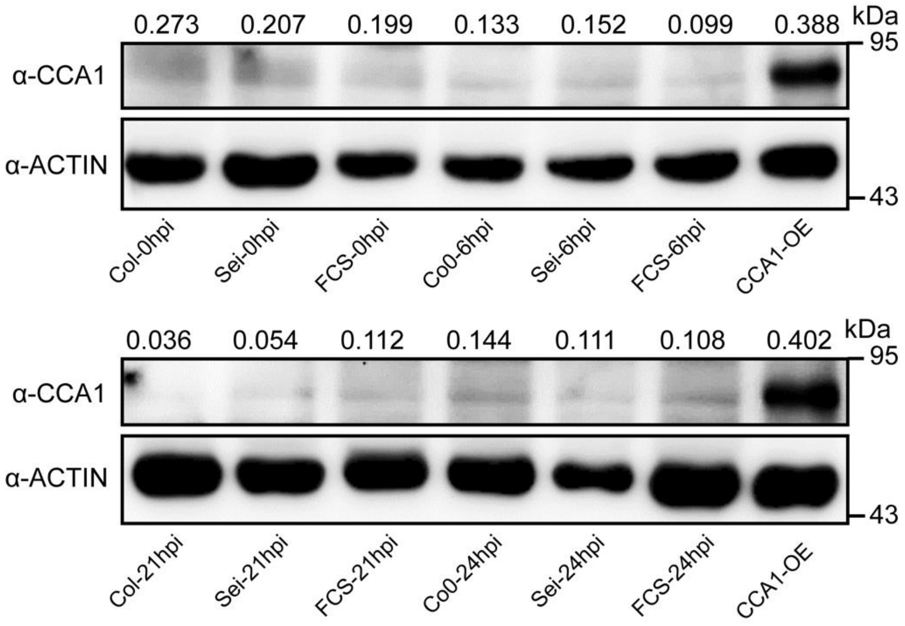

Supplementary Fig. 15. CCA1 proteins were differentially expressed between the FCS hybrids and parents at both 6 hpi and 21 hpi.

Numbers above the bands indicated the intensities of CCA1 protein normalized to ACTIN, which calculated by image J software. CCA1-OE was used as a positive control. ACTIN was used as a loading control.

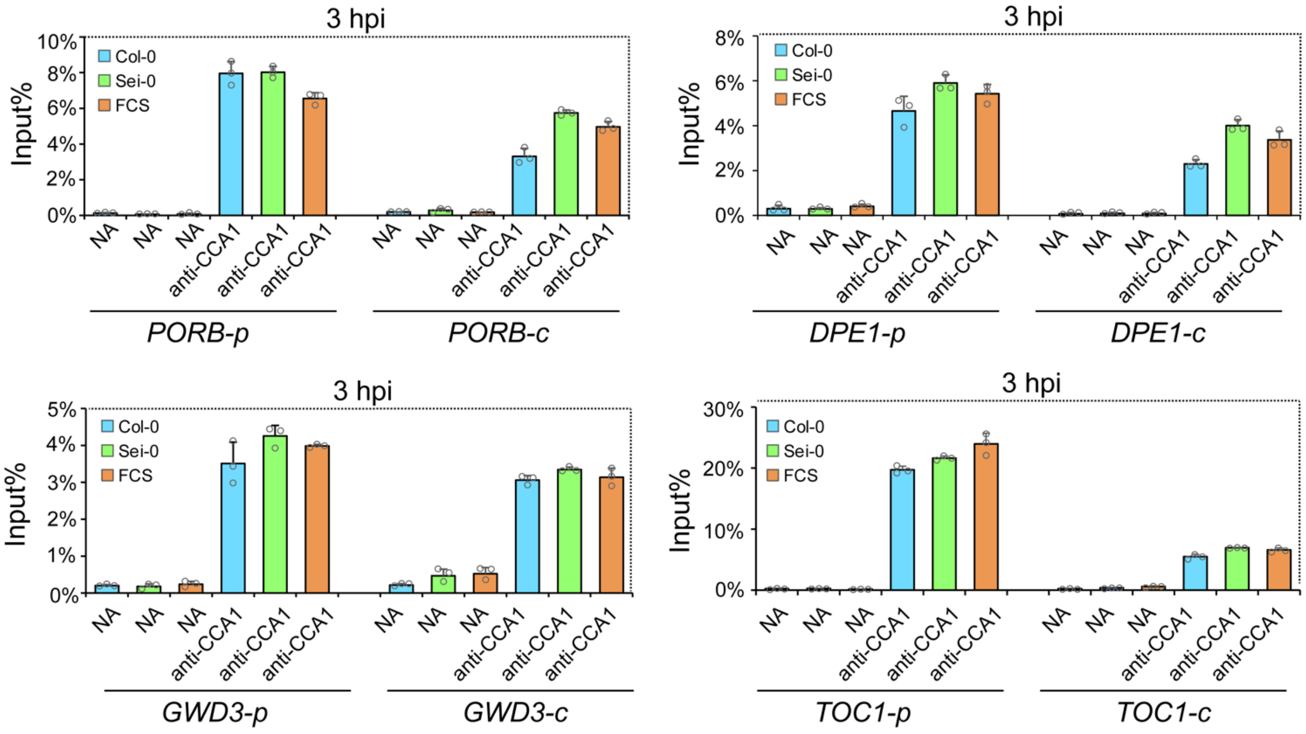

Supplementary Fig. 16. Enrichment of *CCA1* on the promoter of chlorophyll biosynthetic and starch degradation genes showed no difference between the FCS hybrids and parents at 3 hpi.

ChIP-qPCR analyses of promoter fragments that contained 'evening element' motif (*PORB-p*, *GWD3-p*, *DPE1-p* and *TOC1-p*) and exon fragments (*PORB-c*, *GWD3-c*, *DPE1-c* and *TOC1-c*) of *PORB*, *GWD3*, *DPE1* and *TOC1* in F_1 hybrids and their parents using an anti-CCA1 antibody at 3 hpi. The ChIP values were normalized to their respective DNA inputs. The results are representative of three independent experiments, with measurements that were taken from independent samples grown and processed at different times. Data are shown as the mean ± SD (n = 3, n indicates biological replicates). hpi, hours post inoculation.

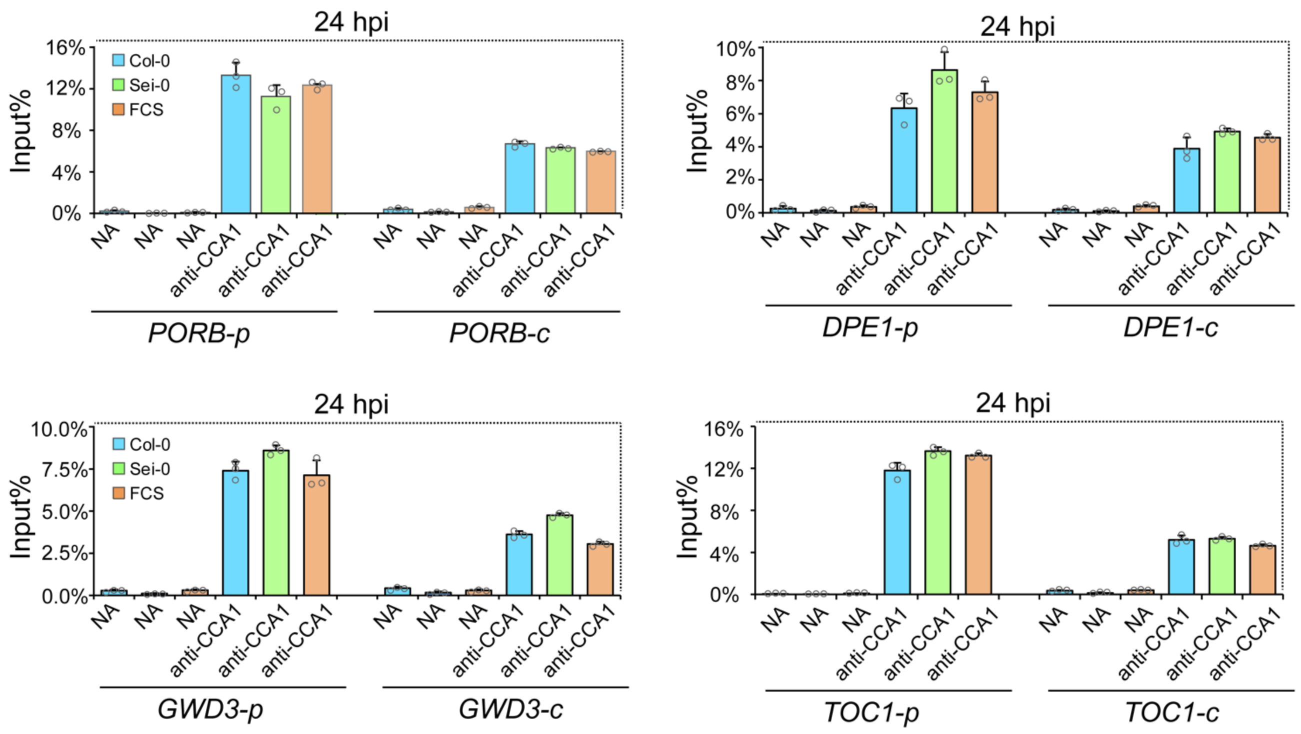

Supplementary Fig. 17. Enrichment of *CCA1* on the promoter of chlorophyll biosynthetic and starch degradation genes showed no difference between the FCS hybrids and parents at 24 hpi.

ChIP-qPCR analyses of promoter fragments that contained 'evening element' motif (*PORB-p*, *GWD3-p*, *DPE1-p* and *TOC1-p*) and exon fragments (*PORB-c*, *GWD3-c*, *DPE1-c* and *TOC1-c*) of *PORB*, *GWD3*, *DPE1* and *TOC1* in F_1 hybrids and their parents using an anti-CCA1 antibody at 24 hpi. ChIP values were normalized to their respective DNA inputs. The results are representative of three independent experiments, with measurements that were taken from independent samples grown and processed at different times. Data are shown as the mean ± SD (n = 3, n indicates biological replicates). hpi, hours post inoculation.

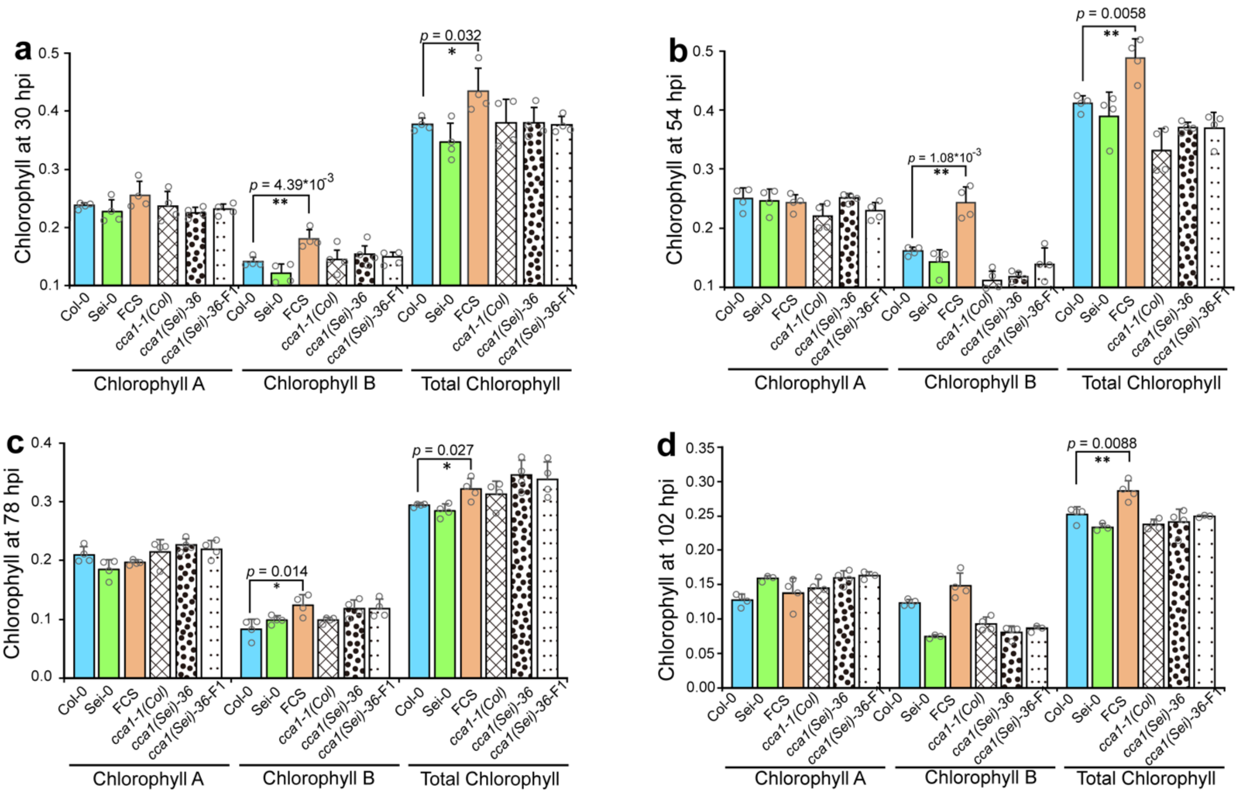

Supplementary Fig. 18. Enhanced accumulation of chlorophyll in hybrids occurred rhythmically in the middle of each infection day.

a-**d** Chlorophyll content (mg/g) of inoculated leaves in F_1 hybrids and parents of the Col-0 × Sei-0 and *cca1-1(Col)* × *cca1(sei)-36* at 30 (**a**), 54 (**b**), 78 (**c**) and 102 (**d**) hours post infiltration with *Pst* DC3000. Data are shown as the mean ± SD (n = 4, n indicates biological replicates, 6 leaves for each biological replicate). hpi, hours post inoculation. '*' *p* value < 0.05 and '**' *p* value < 0.01 (two-tailed Student's *t test*).

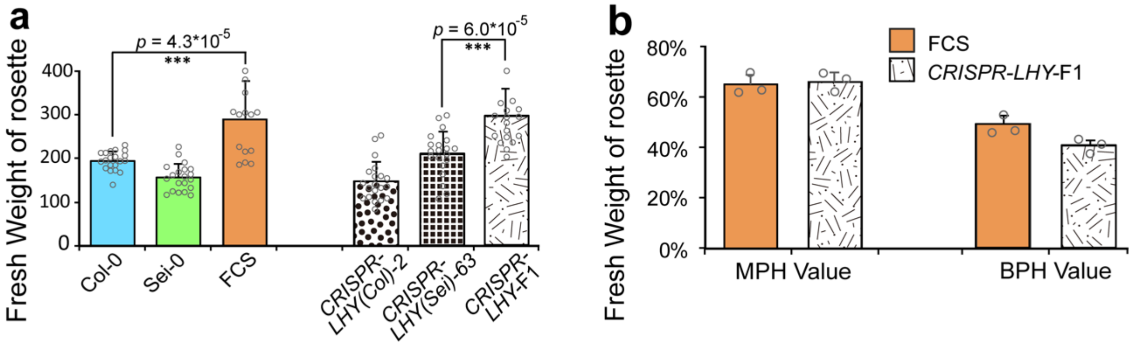

Supplementary Fig. 19. Mutation of *LHY* did not influence the growth heterosis of FCS hybrids.

a Fresh weight of the whole rosette of wild-type and *LHY*-mutated F_1 hybrids and parents at 5 days post infiltration with *Pst* DC3000. Data are shown as the mean ± SD (n = 30 plants for each genotype). **b** Mid-parent heterosis (MPH) value and Best-parent heterosis (BPH) value calculated by whole rosette fresh weight at 5 days post infiltration with *Pst* DC3000 for wild-type and *LHY*-mutated F_1 hybrids. '***' *p* value < 0.001 (two-tailed Student's *t test*). Data are shown as the mean ± SD. The results in **b** are representative of three independent experiments, with measurements that were taken from independent samples grown and processed at different times.

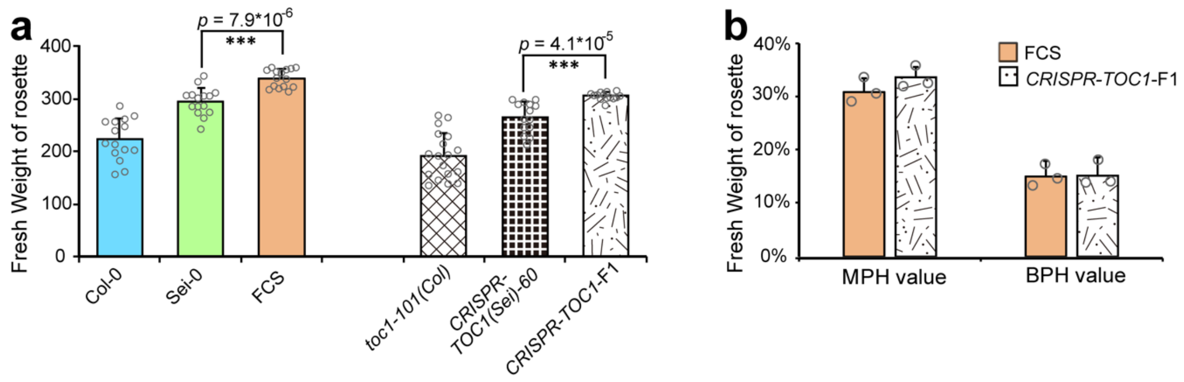

Supplementary Fig. 20. Mutation of *TOC1* did not influence the growth heterosis of FCS hybrids.

a Fresh weight of whole rosette of wild-type and *TOC1*-mutated F_1 hybrids and parents at 5 days post infiltration with *Pst* DC3000. Data are shown as mean ±SD (n = 30 plants for each genotype). '***' *p* value < 0.001 (two-tailed Student's *t test*). **b** Mid-parent heterosis (MPH) and Best-parent heterosis (BPH) values calculated by the whole rosette fresh weight at 5 days post infiltration with *Pst* DC3000 for the wild-type and *TOC1*-mutated F_1 hybrids. Data are shown as the mean ±SD. The results in **b** are representative of three independent experiments, with measurements that were taken from independent samples grown and processed at different times.

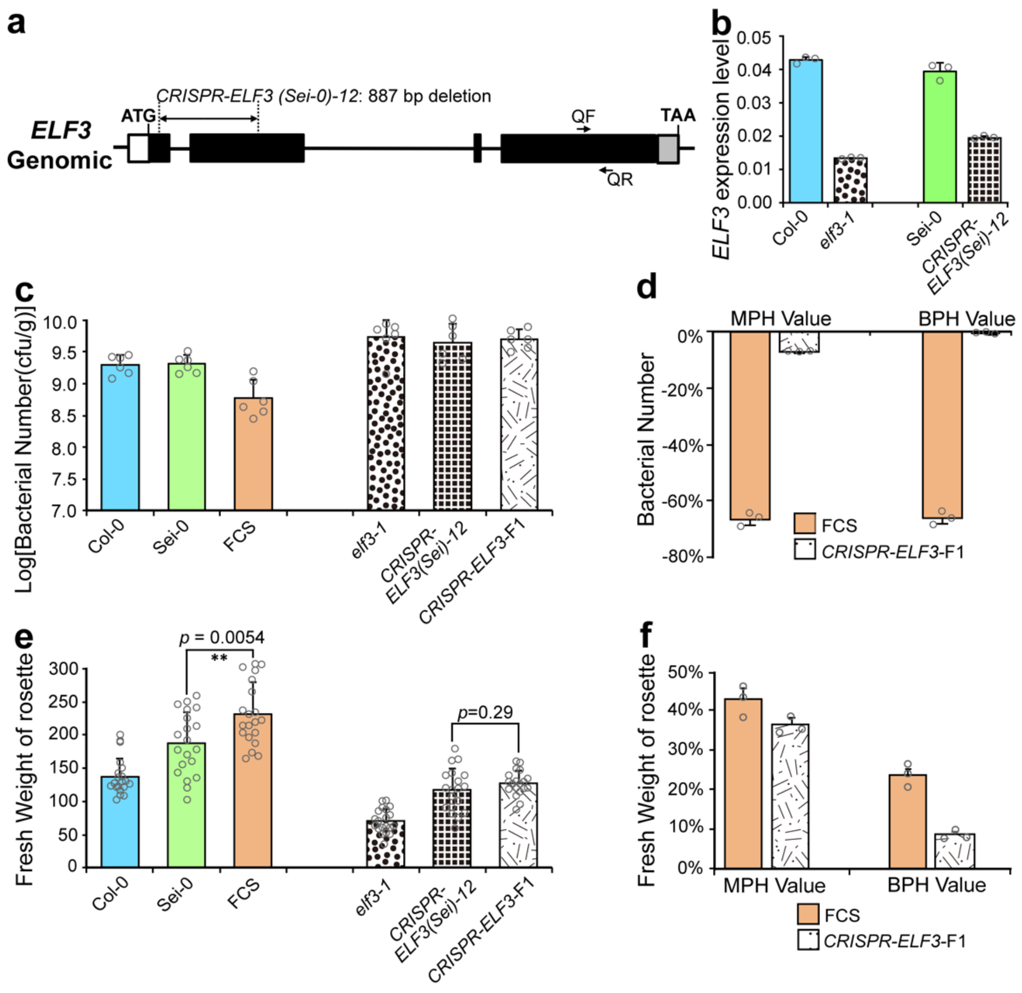

Supplementary Fig. 21. Mutation of *ELF3* significantly decreases both defence and growth heterosis in FCS hybrids.

a Gene model of *ELF3-CRISPR* mutants in the Sei-0 background. White box, promoter; black boxes, exons; gray boxes, 3'UTR. Lines with arrowheads indicate the fragment deleted in *CRISPR* mutants. Primers used to test the transcript level of *ELF3* are indicated by arrows. **b** The transcript level of *ELF3* mutants and wild-type at ZT12. The transcript level of *ELF3* (ZT12, 12-day-old seedlings) is shown as the mean ± SD (n = 3, n indicates biological replicates) and standardized for the abundance of the *ACTIN2* transcript. **c** Bacterial titer (log10) of the wild-type and *ELF3*-mutated F_1 hybrids and parents at 5 days post infiltration with *Pst* DC3000. Bacterial growth is expressed as the mean values of viable bacteria per gram of leaf tissue ± SD (n = 6, n indicates biological replicates). **d** Mid-parent heterosis (MPH) and Best-parent heterosis (BPH) values calculated by bacterial number at 5 days post infiltration with *Pst* DC3000 for wild-type

and *ELF3*-mutated F_1 hybrids. Data are shown as the mean ± SD. The results in **c** and **d** are representative of three independent experiments, with measurements that were taken from independent samples grown and processed at different times. **e** Fresh weight of the whole rosette of wild-type and *ELF3*-mutated F_1 hybrids and parents at 5 days post infiltration with *Pst* DC3000. Data are shown as the mean ± SD (n = 30 plants for each genotype). '**' $p < 0.01$ (two-tailed Student's *t test*). **f** MPH and BPH values calculated by the whole rosette fresh weight at 5 days post infiltration with *Pst* DC3000 for the wild-type and *ELF3*-mutated F_1 hybrids. Data are shown as the mean ± SD. The results in **f** are representative of three independent experiments, with measurements that were taken from independent samples grown and processed at different times.

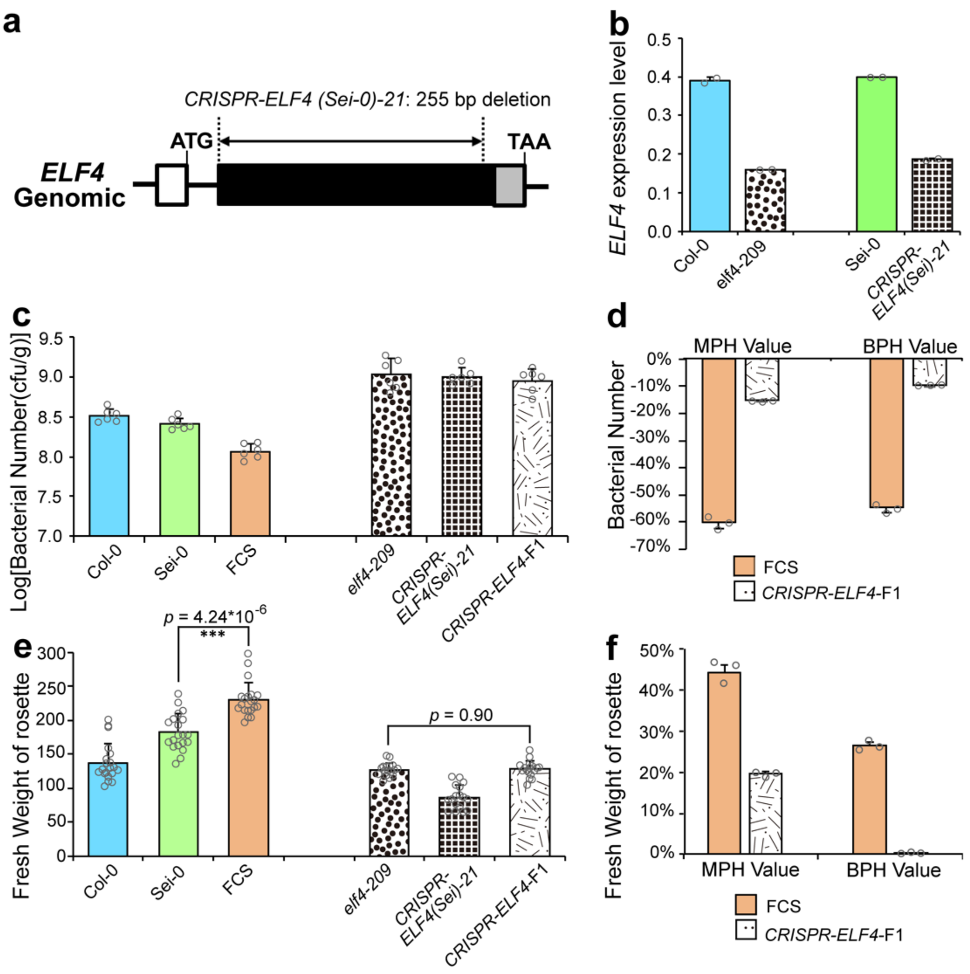

Supplementary Fig. 22. Mutation of *ELF4* significantly decreases both defence and growth heterosis of FCS hybrids.

a Gene model of *ELF4-CRISPR* mutants in Sei-0 background. White box, promoter; black boxes, exons; gray boxes, 3'UTR. Lines with arrowheads indicate the fragment deleted in *CRISPR* mutants. Primers used to test the transcript level of *ELF4* are indicated by arrows. **b** The transcript level of *ELF4-CRISPR* mutants and wild-type at ZT12. The transcript level of *ELF4* (ZT12, 12-day-old seedlings) was shown as the mean ± SD (n = 3, n indicates biological replicates) and standardized for the abundance of the *ACTIN2* transcript. **c** Bacterial titer (log10) of the wild-type and *ELF4*-mutated F_1 hybrid and parents at 5 days post infiltration with *Pst* DC3000. Bacterial growth is expressed as the mean values of viable bacteria per gram of leaf tissue ± SD (n = 6, n indicates biological replicates). **d** Mid-parent heterosis (MPH) and Best-parent heterosis (BPH) values calculated by bacterial number at 5 days post infiltration with *Pst* DC3000

for wild-type and *ELF4*-mutated F_1 hybrids. Data are shown as the mean ± SD. The results in **c** and **d** are representative of three replicates, with measurements that were taken from independent samples grown and processed at different times. **e** Fresh weight of the whole rosette of wild-type and *ELF4*-mutated F_1 hybrids and parents at 5 days post infiltration with *Pst* DC3000. Data are shown as mean ± SD (n = 30 plants for each genotype). '***' p value < 0.001 (two-tailed Student's *t test*). **f** MPH and BPH values calculated by the whole rosette fresh weight at 5 days post infiltration with *Pst* DC3000 for the wild-type and *ELF4*-mutated F_1 hybrids. Data are shown as the mean ± SD. The results in **f** are representative of three independent experiments, with measurements that were taken from independent samples grown and processed at different times.

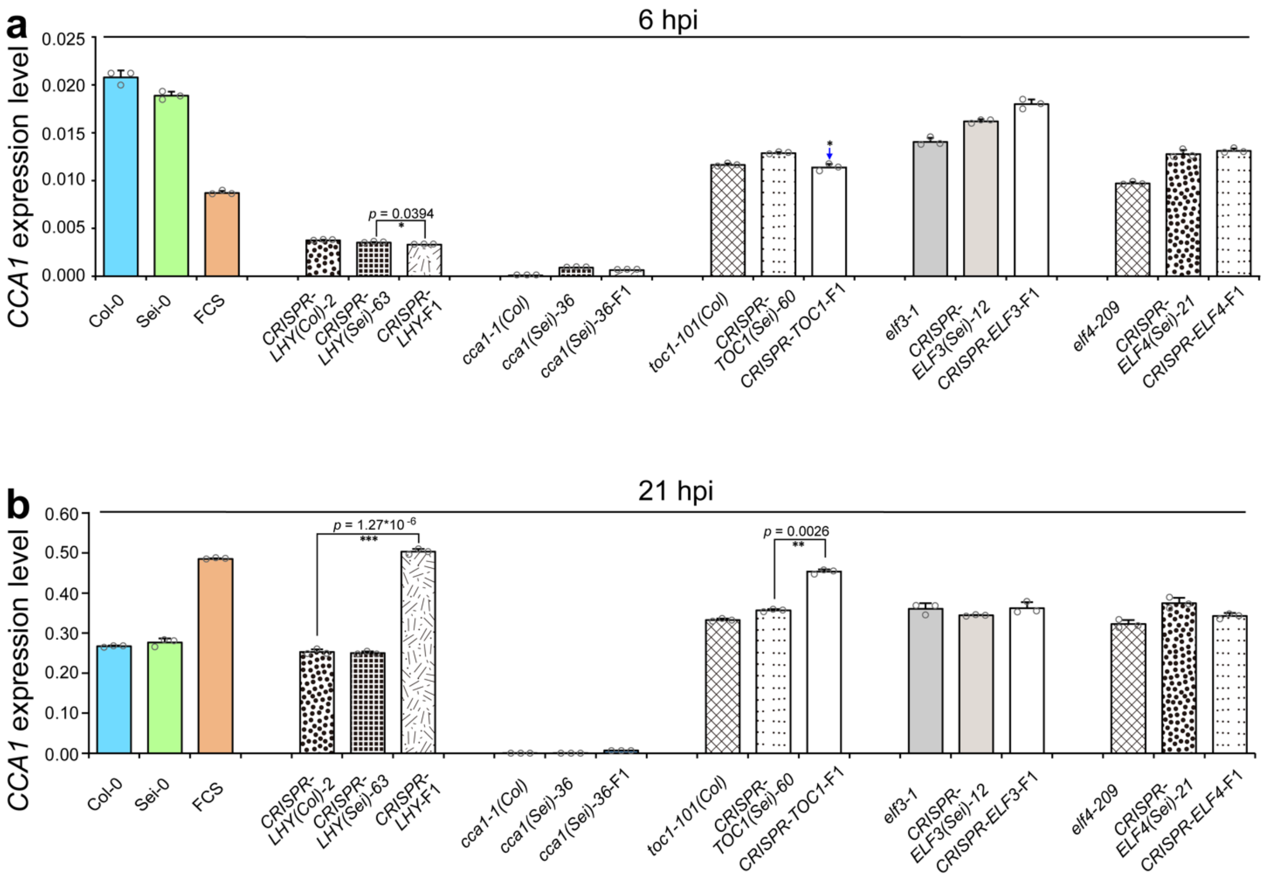

Supplementary Fig. 23. The expression level of *CCA1* in the wild-type and *LHY*, *TOC1*, *ELF3*, and *ELF4*-mutated parents and hybrids at 6 and 21 hpi.

Quantitative RT-PCR analysis of *CCA1*'s expression level in FCS hybrid, *LHY*, *TOC1*, *ELF3*, *ELF4*-mutated F_1 hybrids, and their parents (*ACTIN2* as a control) at 6 hpi (**a**) and 21 hpi (**b**). Data are shown as the mean ± SD (n = 3, n indicates biological replicates). Arrows indicate significant down-(blue) regulation of *CCA1* in F_1 hybrids compared with the mid-parent value (MPV). hpi, hours post inoculation. '**' *p* value < 0.01; '***' *p* value < 0.001 (two-tailed Student's *t test*).

Supplementary Tables:

Supplementary Table 1. Genes for GO analysis in Supplementary Figure 1.

AT1G62430	AT4G39190	AT4G23210	AT2G22450	AT3G43790
AT2G22970	AT2G33150	AT5G40720	AT3G04550	AT2G45850
AT3G16565	AT1G07040	AT5G01550	AT1G70640	AT2G04860
AT4G31290	AT4G23550	AT1G18570	AT4G26530	AT2G22830
AT4G13250	AT2G28080	AT3G53065	AT3G10520	AT2G37220
AT4G13340	AT1G11210	AT2G30395	AT5G26570	AT5G39210
AT1G10200	AT1G49720	AT3G04060	AT1G28050	AT5G27820
AT1G11530	AT3G16180	AT1G63720	AT1G29670	AT5G44260
AT3G27300	AT5G41610	AT1G44130	AT5G48250	AT1G51610
AT4G39260	AT1G17190	AT4G19970	AT2G21660	AT4G09020
AT5G30495	AT3G26740	AT1G53625	AT1G68050	AT3G53900
AT4G12290	AT4G17670	AT5G06690	AT5G14320	AT1G69040
AT5G17460	AT4G24690	AT1G53100	AT1G80770	AT1G54410
AT1G68820	AT4G33985	AT3G47800	AT4G39460	AT3G18680
AT3G59350	AT3G46440	AT1G52200	AT4G16390	AT3G42670
AT2G18170	AT2G15960	AT2G15890	AT1G29070	AT1G33770
AT4G33920	AT4G27440	AT2G33830	AT1G79460	AT4G30660
AT2G27660	AT2G01818	AT3G13610	AT3G20230	AT4G26370
AT1G67970	AT4G25480	AT1G48700	AT1G74670	AT1G78460
AT4G12480	AT4G21830	AT2G15090	AT5G03940	AT4G36180
AT1G02450	AT1G19370	AT5G23020	AT1G08660	AT4G31050
AT1G21680	AT1G65690	AT3G05130	AT3G28860	AT1G20030
AT3G50970	AT5G26340	AT3G46970	AT3G24430	AT1G48330
AT3G05880	AT2G27200	AT2G29630	AT2G02070	AT2G30424
AT1G61800	AT3G28340	AT5G51820	AT1G77210	AT5G24470
AT5G05410	AT4G04330	AT4G19120	AT5G04360	AT4G25490
AT5G40010	AT4G16146	AT4G28140	AT1G05190	AT3G53800
AT4G12500	AT4G14270	AT3G10020	AT4G16141	
AT2G16700	AT4G00700	AT1G10760	AT2G15970	
AT4G30190	AT1G79450	AT5G11150	AT3G15570	

Supplementary Table 2. Degree of defence heterosis at 5 dpi and the *CCA1*'s differential expression pattern at 21 hpi in 18 hybrids (Col-0 was the maternal line).

F_1 Hybrids	Paternal line stock Number	Paternal line Longitude/Latitude	MPH for bacterial number (5 days post infiltration with *Pst* DC3000)	MPH for *CCA1* exp. at 21 hpi
Col-0 × Wl-0	CS76630	10.81/47.92	179.42%	-19.38%
Col-0 × Rue3.1-31	CS76406	9.16/48.56	363.87%	-35.68%
Col-0 × Ema-1	CS76480	0.50/51.3	64.08%	-32.83%
Col-0 × Koch-1	CS76396	29.32/50.36	62.14%	-36.84%
Col-0 × WalhaesB4	CS76408	9.19/48.60	150.88%	-22.83%
Col-0 × Ca-0	CS76459	8.27/50.30	123.2%	-1.87%
Col-0 × Est	CS76485	24.99/58.67	28.82%	-21.98%
Col-0 × Aa-0	N934	9.57/50.92	128.83%	-27.60%
Col-0 × ICE107	CS76364	16.22/38.38	-87.23%	-36.65%
Col-0 × NC-1	CS76559	6.25/8.62	-27.59%	-5.31%
Col-0 × Bd-0	CS76445	13.29/52.46	-75.54%	<u>18.08%</u>
Col-0 × Ak-1	CS76431	7.63/48.07	-60.02%	<u>16.74%</u>
Col-0 × ICE91	CS76362	16.17/38.62	-34.82%	-32.72%
Col-0 × Ba-1	CS76441	-4.80/56.55	-75.95%	<u>40.74%</u>
Col-0 × Altai-5	CS76433	88.40/47.75	-80.14%	-49.40%
Col-0 × Bay-0	CS955	11.00/49.00	-90.05%	-33.63%
Col-0 × Ber	CS76448	12.00/55.00	-89.08%	<u>21.47%</u>
Col-0 × *Ler*-1	CS6928	10.87/47.98	-76.90%	<u>17.01%</u>

MPH, <u>m</u>id-<u>p</u>arent <u>h</u>eterosis.

Supplementary Table 3. Degree of growth heterosis at 5 dpi and the *CCA1*'s differential expression pattern at 6 hpi in 14 hybrids (Col-0 was the maternal line).

F_1 Hybrids	MPH for Fresh weight (5 days post infiltration with *Pst* DC3000)	BPH for Fresh weight (5 days post infiltration with *Pst* DC3000)	MPH for *CCA1* exp. at 6 hpi
Col-0 × Ak-1	97.07%	91.07%	-40.22%
Col-0 × NC-1	46.45%	40.59%	-13.31%
Col-0 × Est	84.44%	76.44%	-9.10%
Col-0 × Bd-0	116.33%	85.80%	-40.01%
Col-0 × Ca-0	-2.78%	2.90%	-22.94%
Col-0 × ICE107	69.55%	78.69%	-20.80%
Col-0 × Altai-5	82.93%	69.16%	-8.71%
Col-0 × Rue3.1-31	35.76%	33.95%	-19.78%
Col-0 × Bay-0	61.86%	59.14%	-21.93%
Col-0 × Ema-1	95.47%	90.78%	-24.41%
Col-0 × WalhaesB4	41.18%	47.35%	27.41%
Col-0 × Koch-1	63.90%	74.06%	5.54%
Col-0 × Wl-0	26.66%	29.63%	14.94%
Col-0 × ICE91	103.13%	115.73%	38.39%

MPH, mid-parent heterosis; BPH, best-parent heterosis.

Supplementary Table 4. Primers used in this study.

Experiment	Name	Sequence
**qRT-PCR**		
CCA1	F	CCTCGTCAGACACAGACTTCCA
	R	CCGCAGTAGAATCAGCTCCAATA
PR1	F	CTCATACACTCTGGTGGG
	R	TTGGCACATCCGAGTC
CBP60g	F	AATAACGAGGAGGATGAGAACG
	R	TCAGACACGGTAAGAAACATCG
SARD1	F	CCTCAACCAGCCCTACGTTA
	R	TAGTGGCTCGCAGCATATTG
PAD4	F	ACCGAGGAACATCAGAGGTAC
	R	AAATTCGCAATGTCGAGTGGC
EDS1	F	CAAGAATCTTGAAGCTGTCATTGATC
	R	TGTCCTGTGAACACTATCTGTTTTCTACT
ICS1	F	GAACTCAAATCTCAACCTCC
	R	ACTGCGACGAGAGAAGAAAC
PORA	F	AAACCATTTGGGCCACTTTCTT
	R	CAAGTCTTTTCCCAGCCTCTGA
PORB	F	ACCAAATCAAATCCGAACATGG
	R	GGCTCTTTAGCTGTCGGGAAAT
GWD3	F	GCCATTGTTGCAGCTCTCCTTT
	F	TTCCAACTCACAAACCCATCCA
DPE1	F	CCAAAACCCTGCAAATCCTCTG
	R	AAAGGCAGTGGCAGAAAGTTCG
ACTIN2	F	GTCTGTGACAATGGAACTGGAA
	R	CTTTCTGACCCATACCAACCAT
**ChIP**		
	CBP60g-p-CF	TCACTGCTGCTTCGTCAATAC
	CBP60g-p-CR	CTAGGGCTGTTCCGAATCTTCATTG
	CBP60g-c-CF	AGTGATGAGGTTTGGAGACTAGA
	CBP60g-c-CR	ACGTTGTGTAACTCATTTCG
	SARD1-p-CF	GATTATTCGCGTGGATCAGACTTCG
	SARD1-p-CR	CTGCCATGGAATTGTTCTGGTGA
	SARD1-c-CF	TAGACATCGTTGCTCTTCAC
	SARD1-c-CR	CTCGAATTGTCTGTGAACAC
	PAD4-p-CF	GATCACATGCTTTGATTCGC
	PAD4-p-CR	GTCGTCTTCTTCAAAGTCTC
	PAD4-c-CF	GGCTAAGCTTGAGCAAGCAA
	PAD4-c-CR	CGAGTTCTTCGCTTTAACATCC
	EDS1-p-CF	GTCCACTAAAGAAAAGAGAA
	EDS1-p-CR	AAGATTACGACTGCTCCTGC
	EDS1-c-CF	CTTAGGGTTACAGCTAGAAG
	EDS1-c-CR	CAAGCATCCCTTCTAATGTC
	PORB-p-CF	GTATGGAATCCCATCAACACC

	PORB-p-CR	ACAGAGGTCACCGAAGATGT
	PORB-c-CF	CAGCTCAGCTATGATTGATGG
	PORB-c-CR	GAGAGGAATGTGCTCTCGGAA
	GWD3-p-CF	GCGAAACTCTCTATAATTGATTG
	GWD3-p-CR	GTTAACGAGTCTAGGAAGTG
	GWD3-c-CF	GAAGCATCTCCAAGTCATGTG
	GWD3-c-CR	GGAGGCTATTGGAAGATGAG
	TOC1-p-CF	TTTTATGGCCTGCACTTTTTATTG
	TOC1-p-CR	GGTGGGACTTGGGATATTTTAGG
	TOC1-c-CF	GCTACAGCCAAAAAAACATCGA
	TOC1-c-CR	GAGCCGCAAGAGCCAACAT
	DPE1-p-CF	CACGAGAGTTTCCTTACACC
	DPE1-p-CR	GACGGCCTAAGTAGAATCGA
	DPE1-c-CF	CCCTCTTTATGACTGGAAAGC
	DPE1-c-CR	ATAATGACTTTCCAGGTCCTAC
	CCA1-CF1	GTGTTGTAAATTCCTCAAGAC
	CCA1-CR1	GCATAAGGCCCATATCGTTATTC
	CCA1-CF2	CTCCATTTCCGTAGCTTCTGG
	CCA1-CR2	CAAACAATAAGAAAGACCATGAC
	CCA1-CF3	GATCTGAAGTTGTGTAGAGGAG
	CCA1-CR3	GGCTTCCGAGTCTATATCAAG
	CCA1-CF4	CTCAAGCTTCCACATGAGACTC
	CCA1-CR4	GTTACAGGAAGACTATGGACAAG
**Plasmid construction**		
***CCA1* CRISPR**	CCA1-T1-DT1-BsF	ATATATGGTCTCGATTGCAACGTGAAAGGTGGACTGGTT
	CCA1-T1-DT1-F0	TGCAACGTGAAAGGTGGACTGGTTTTAGAGCTAGAAATAGC
	CCA1-T6-DT2-R0	AACCCACTAGAATCGGGAGGCCCAATCTCTTAGTCGACTCTAC
	CCA1-T6-DT2-BsR	TTATTGGTCTCGAAACCCACTAGAATCGGGAGGCCC
***LHY* CRISPR**	LHY-T1-DT1-BsF	ATATATGGTCTCGATTGCAGCGAGAGCGATGGACTGGTT
	LHY-T1-DT1-F0	TGCAGCGAGAGCGATGGACTGGTTTTAGAGCTAGAAATAGC
	LHY-T3-DT2-R0	AACCGTTGTCCACAGTTGAGGTCAATCTCTTAGTCGACTCTAC
	LHY-T3-DT2-BsR	ATTATTGGTCTCGAAACCGTTGTCCACAGTTGAGGTC
***TOC1* CRISPR**	TOC1-T2-DT1-BsF	ATATATGGTCTCGATTGTGAACGGTGAGTGTAAAGGGTT
	TOC1-T2-DT1-F0	TGTGAACGGTGAGTGTAAAGGGTTTTAGAGCTAGAAATAGC
	TOC1-T3-DT2-R0	AACCCAAACCCCGATGAAGATGCAATCTCTTAGTCGACTCTAC
	TOC1-T3-DT2-BsR	ATTATTGGTCTCGAAACCCAAACCCCGATGAAGATGC
***ELF3* CRISPR**	ELF3-T1-DT1-BsF	ATATATGGTCTCGATTGGCTCCTCCTAGAAACAAGAGTT
	ELF3-T1-DT1-F0	TGGCTCCTCCTAGAAACAAGAGTTTTAGAGCTAGAAATAGC
	ELF3-T2-DT2-R0	AACCTGTGACTGATACATCTAACAATCTCTTAGTCGACTCTAC
	ELF3-T2-DT2-BsR	ATTATTGGTCTCGAAACCTGTGACTGATACATCTAAC
***ELF4* CRISPR**	ELF4-T1-DT1-BsF	ATATATGGTCTCGATTGGGGAGAGGATCCGGCGATGGTT
	ELF4-T1-DT1-F0	TGGGGAGAGGATCCGGCGATGGTTTTAGAGCTAGAAATAGC
	ELF4-T2-DT2-R0	AACCCACCATCGTGACCGTTCTCAATCTCTTAGTCGACTCTAC
	ELF4-T2-DT2-BsR	ATTATTGGTCTCGAAACCCACCATCGTGACCGTTCTC
**Mutant screening and identification**		
**CCA1**	CCA1-Crispr-IDF	CATTGAATCATCTCTCTAAAGG
	CCA1-Crispr-IDR	CATTCGTTTCTTTCACCTCA

	CCA1-Crispr-ID-QF	CCTCGTCAGACACAGACTTCCA
	CCA1-Crispr-ID-QR	CCGCAGTAGAATCAGCTCCAATA
**LHY**	LHY-Crispr-IDF	GAGAGGATTTGAAGCAGCGA
	LHY-Crispr-IDR	TGAGGGATGATTTTGAGGGC
	LHY-Crispr-ID-QF	CAGGATGATTACCGTTCGTTTC
	LHY-Crispr-ID-QR	GCAATGGCAGTTATACTTGGAG
**TOC1**	TOC1-Crispr-IDF	CTGATTTGGCCATGGAAGTA
	TOC1-Crispr-IDR	CTGTTCATAGCAACTTGGGG
	TOC1-Crispr-ID-QF	GTTGATGGATCGGGTTTCTC
	TOC1-Crispr-ID-QR	TCATGACCCCATGCATACAG
**ELF3**	ELF3-Crispr-IDF	GTGGTGAGATCTCTATCGTAG
	ELF3-Crispr-IDR	CTACCGAGGACTCTACAACT
	ELF3-Crispr-ID-QF	CAGCAATGGTTGATCCCTGT
	ELF3-Crispr-ID-QR	GAGGGAAGTAGCCATTACCA
**ELF4**	ELF4-Crispr-IDF	GTGTCCGATTCTACTCAGAAG
	ELF4-Crispr-IDR	AGCTTGAACCGGACAGATTC
	ELF4-Crispr-ID-QF	CTGCCGGAACTAGAGCTTAA
	ELF4-Crispr-ID-QR	CCACGGATTATTCTAACGAC
**Cas9**	pHEE401-Cas9-IDF	TCGAGAGCGAGTTCGTGTAC
	pHEE401-Cas9-IDR	TTCGGTCATTAGAGGCCACG
