# Supplementary material for: A central circadian oscillator confers defense heterosis in hybrids without growth vigor costs
Source: Nat Commun. 2021 Apr 19;12:2317. doi: 10.1038/s41467-021-22268-z (PMC8055661; doi:10.1038/s41467-021-22268-z)
Supplement: Supplementary file 2 — Descriptions of Additional Supplementary Files [file 41467_2021_22268_MOESM2_ESM.docx]

Descriptions of Additional Supplementary Files

**Supplementary Data 1**

**Description:** Above-high parent DEGs for motif enrichment
